# Understanding perceived availability and importance of tobacco control interventions to inform European adoption of a UK economic model: a cross-sectional study

**DOI:** 10.1186/s12913-018-2923-2

**Published:** 2018-02-14

**Authors:** Puttarin Kulchaitanaroaj, Zoltán Kaló, Robert West, Kei Long Cheung, Silvia Evers, Zoltán Vokó, Mickael Hiligsmann, Hein de Vries, Lesley Owen, Marta Trapero-Bertran, Reiner Leidl, Subhash Pokhrel

**Affiliations:** 1 Health Economics Research Group (HERG), Institute of Environment, Health and Societies, Brunel University London, Uxbridge, UK; 2 Syreon Research Institute, Budapest, Hungary; 3 Department of Health Policy & Health Economics, Faculty of Social Sciences, Eötvös Loránd University, Budapest, Hungary; 4 Health Behaviour Research Centre, University College London, London, UK; 5 National Centre for Smoking Cessation and Training, Birmingham, UK; 6 Department of Health Promotion, Caphri School of Public Health, Maastricht University, Maastricht, the Netherlands; 7 Department of Health Services Research, Caphri School of Public Health, Maastricht University, Maastricht, the Netherlands; 8 National Institute for Health and Care Excellence, London, UK; 9 Centre for Research in Economics and Health, University Pompeu Fabra, Barcelona, Spain; 10 Institute of Health Economics and Healthcare Management, Helmholtz Zentrum München, Oberschleißheim, Germany

**Keywords:** Tobacco control, Smoking cessation, Evidence transferability, Economic model

## Abstract

**Background:**

The evidence on the extent to which stakeholders in different European countries agree with availability and importance of tobacco-control interventions is limited. This study assessed and compared stakeholders’ views from five European countries and compared the perceived ranking of interventions with evidence-based ranking using cost-effectiveness data.

**Methods:**

An interview survey (face-to-face, by phone or Skype) was conducted between April and July 2014 with five categories of stakeholders - decision makers, service purchasers, service providers, evidence generators and health promotion advocates - from Germany, Hungary, the Netherlands, Spain, and the United Kingdom. A list of potential stakeholders drawn from the research team’s contacts and snowballing served as the sampling frame. An email invitation was sent to all stakeholders in this list and recruitment was based on positive replies. Respondents were asked to rate availability and importance of 30 tobacco control interventions. Kappa coefficients assessed agreement of stakeholders’ views. A mean importance score for each intervention was used to rank the interventions. This ranking was compared with the ranking based on cost-effectiveness data from a published review.

**Results:**

Ninety-three stakeholders (55.7% response rate) completed the survey: 18.3% were from Germany, 17.2% from Hungary, 30.1% from the Netherlands, 19.4% from Spain, and 15.1% from the UK. Of those, 31.2% were decision makers, 26.9% evidence generators, 19.4% service providers, 15.1% health-promotion advocates, and 7.5% purchasers of services/pharmaceutical products. Smoking restrictions in public areas were rated as the most important intervention (mean score = 1.89). The agreement on availability of interventions between the stakeholders was very low (kappa = 0.098; 95% CI = [0.085, 0.111] but the agreement on the importance of the interventions was fair (kappa = 0.239; 95% CI = [0.208, 0.253]). A correlation was found between availability and importance rankings for stage-based interventions. The importance ranking was not statistically concordant with the ranking based on published cost-effectiveness data (Kendall rank correlation coefficient = 0.40; *p*-value = 0.11; 95% CI = [− 0.09, 0.89]).

**Conclusions:**

The intrinsic differences in stakeholder views must be addressed while transferring economic evidence Europe-wide. Strong engagement with stakeholders, focussing on better communication, has a potential to mitigate this challenge.

**Electronic supplementary material:**

The online version of this article (10.1186/s12913-018-2923-2) contains supplementary material, which is available to authorized users.

## Background

The human and economic costs due to adverse effects of tobacco use have shown to be enormous-- about 700,000 lives claimed [1] and estimated annual economic burden of at least €100 billion annually in Europe [2]. Even though effort has been made in tobacco control, smoking is still the largest single cause of death and diseases in Europe [2]. Co-ordinated, high impact and comprehensive approaches are consistently shown to be the most effective way to reduce smoking initiation, prevalence, and intensity of consumption [3–6]. However, generation of research evidence on those comprehensive approaches often occur in certain countries [3, 7]. It is not clear to what extent that evidence could be transferable to other settings where resources to conduct similar research are scarce [8–14].

In the United Kingdom (UK), a decision-support tool (the National Institute for Health and Care Excellence (NICE) Tobacco Return on Investment (ROI) tool) providing the information about economic and wider returns from evidence-based tobacco control interventions was developed for policy makers and wider groups of stakeholders [15]. There was an aim to further develop this tool to be used in other European countries in a large comparative effectiveness research study, namely European-study on Quantifying Utility of Investment in Protection from Tobacco (EQUIPT) [16]. EQUIPT sought to first understand pre-requisites such as contextual realities in new settings when transferring the decision-support tool to sample European countries: Germany, Hungary, the Netherlands, and Spain [16]. The sample countries were selected because they are from five European Union (EU) member states with significant differences in population health outcomes, prevalence of smoking, economic status, and health care spending [2]. This cross-section was deemed to be broadly representative of the EU countries.

To transfer evidence or an economic model, the need to understand the contextual realities of a new setting is highlighted in the literature [17, 18]. This involves appraisal of applicability (i.e. the extent to which the evidence can be used in a new setting) and transferability (i.e. the extent to which similar outcomes can be achieved in a new setting) [17, 18]. The appraisal of applicability and transferability should involve stakeholders such as policy-makers, practitioners, and scientists to provide opinion on attributes of applicability and transferability [18]. Such attributes may include availability of essential resources (e.g. whether the intervention is currently available) and political/social acceptability of the intervention (e.g. whether the intervention is important to the stakeholders) [19].

In addition to opinion on availability and importance of tobacco-control interventions, previous research shows that not all stakeholders in the new context interpret intervention descriptions and evaluation findings in the same way, even when the same information is presented [20]. This necessitates an enquiry to better understand stakeholders’ views on importance of tobacco-control interventions in the new settings relative to their reported cost-effectiveness.

The specific objectives of this study therefore were as follows: a) to understand stakeholders’ views about what tobacco control interventions are available in their countries and how important those interventions are to them, in five European countries – Germany, Hungary, Spain, the Netherlands and the UK; and b) to establish the extent of difference in intervention priorities by comparing perceived importance with the ranking based on published cost-effectiveness data.

## Methods

### Data

Data for this study were drawn from the following two sources: (i) the EQUIPT stakeholder interview survey capturing views on availability and importance of tobacco-control interventions [21]; and (ii) published data on cost-effectiveness of tobacco control interventions [22].

First, a stakeholder interview survey (face-to-face, by phone or Skype) was conducted between April and July 2014 as part of the EQUIPT project (http://equipt.eu) [21, 23]. Further information about the interview survey is documented elsewhere [16, 21, 23]. A list of stakeholders categorized into five groups was drawn by the EQUIPT team from Germany, Hungary, the Netherlands, Spain, and the UK based on their previous knowledge; and then those stakeholders were asked to provide additional names (snowballing). This list served as the sampling frame for the interview survey. The five categories of stakeholders included: decision makers (e.g. senior officials at the department of health or directors of public health services), purchasers of services or pharmaceutical products (e.g. service commissioners or top officials at health insurance funds), professionals or service providers (e.g. leading physicians in smoking cessation, physicians/psychologists, or coordinators of local health programs), evidence generators (e.g. academic and researchers with experience in health-technology-assessments, reimbursement procedures, health care costing, general public health and health promotion research, and specific research to tobacco control and smoking cessation), and advocates of health promotion (e.g. leaders of charities, NGO’s or patient organizations).

A researcher from each country interviewed the stakeholders in the local language and filled out the questionnaire, which was developed originally in English and then translated to each of the local languages. To ensure the consistency of the survey across countries, a training workshop was organised in Maastricht in January 2014, followed by a few online training sessions prior to the interviews. A pilot study was conducted to improve the actual interview survey. The interviews were based on pre-defined questionnaire administered by trained interviewers. Thus, interviewer validity was achieved to a reasonable degree. Interview guide and survey questionnaire are provided in Additional file 1. 

The questionnaire included several questions: stakeholders’ profiles, motivational factors, and stakeholders’ opinion. Perception of availability and importance of tobacco-control interventions were captured by the following two questions, respectively: (a) “Would you tell me whether these [show the list] tobacco control measures and smoking cessation interventions are available in …[name country]…?” with possible answers being ‘yes’, ‘no’, and ‘don’t know’; and (b) “Could you also indicate on a scale from 1 to 3 —1 meaning ‘not important’ and 3 meaning ‘important’ – to what extent you think the following interventions are considered important in addressing smoking behaviour?” Respondents were allowed to state ‘don’t know’ as a response in addition to one of the three numbers (1, 2, and 3). A total of 30 interventions grouped into five categories – 3 pharmacological, 11 behavioural, 3 combined pharmacological and behavioural, 7 nonconventional, and 6 population-level - were shown to stakeholders. The intervention list was informed by a previous review conducted as part of the NICE Tobacco ROI project [15].

Secondly, cost-effectiveness data on the selected interventions was sourced from a published study in the UK by Owen and colleagues who comprehensively reviewed the economic evidence of key public health interventions including tobacco-control [22]. This single review study was selected because it provided summarised cost-effectiveness data on most of the tobacco-control interventions listed in the stakeholder survey questionnaire in a comparable way, and thus allowed a head-to-head comparison between the two.

### Ethics, consent, and permission

Brunel University Research Ethics Committee (UK) reviewed this research and gave full ethical clearance. Respective authorities in other countries - Ethik-Kommission, Bayerische Landesärztekammer (Germany), Egészségügyi Tudományos Tanács, Tudományosés Kutatásetikai Bizottság (Hungary), Medisch-ethische toetsingscommissie (METC) azM/UM (Netherlands), and Parc de SalutMAR - Clinical Research Ethics Committee (Spain) - also provided clearance. Written consent was obtained from all respondents.

### Statistical analyses

Three separate analyses were carried out. Firstly, descriptive analyses with all possible responses were conducted to explore stakeholders’ perceptions of availability and importance of tobacco control interventions in each country and between the countries. Kappa statistics were calculated to evaluate the agreement on the perceived availability with all three possible responses and importance with all four possible responses between stakeholders in each country, from two selected countries, and from all five countries. A kappa statistic or a kappa coefficient is commonly used to measure the agreement between multiple observers; it ranges from − 1 to 1, the latter indicating perfect agreement [24]. If it is less than or equal to 0, there is less or no agreement between raters other than what would occur by chance. Kappa values of 0.01–0.20, 0.21–0.40, 0.41–0.60, 0.61–0.80, and 0.81–0.99 refer to slight agreement, fair agreement, moderate agreement, substantial agreement, and almost perfect agreement, respectively [24]. A significant kappa (*p*-value < 0.05) means that the estimated value of the kappa coefficient significantly differs from zero. [24]. Confidence intervals around kappa coefficient were calculated by a bootstrap method as the computation involved more than three raters [25]. Combined kappa coefficients from all possible responses are reported. Moreover, a comparison of perceived importance when combining ‘somewhat important’ (response 2 on the questionnaire) and ‘important’ (response 3) values between the 30 interventions was presented in a visual display. Such comparison was conducted as part of the decision to choose the interventions for the EQUIPT model.

Secondly, to assess whether stakeholders’ views about availability of interventions were correlated with their views about importance of interventions, a descriptive analysis showing distributions of availability and importance was conducted. This relationship was further analysed using an ordered logistic regression [26]. The ‘don’t know’ responses were excluded from the regression analysis. The model included importance (not important, somewhat important, or important) as the dependent variable and availability (yes or no), countries (the UK as the reference country), stakeholder roles (evidence generators as the reference role), and gender (male as the reference group) as covariates.

Thirdly, a perceived importance mean score for each intervention was computed after excluding the ‘don’t-know’ responses. The 3-point Likert scale response on the question related to perceived importance of an intervention was recoded from [1, 2 3] to [0, 1, 2] respectively. Then the mean score was calculated as the arithmetic average of the responses across all stakeholders. This score was finally used to rank the interventions. The other set of ranking involved scrutiny of the cost-effectiveness data from Owen et al. [22]. To do this, first the median costs per quality adjusted life years (QALYs), the only reported measure in that study, were extracted for the interventions that were in common with those listed in the EQUIPT stakeholder survey. Then, interventions were rank ordered using the lowest median costs per QALY and moving upwards. This ranking was then compared with the ranking based on the stakeholders’ views to determine where similarities or differences occurred. Kendall rank (tau) correlation coefficient was estimated to evaluate the difference between the concordance and the discordance probabilities of the mean-importance-score ranking and the cost-effectiveness-measure ranking. The tau coefficient is normally used when a distribution of a variable is not assumed and a sample size is small [27]. The coefficient ranges from − 1 to 1 meaning complete disagreement and complete agreement between the mean importance score and the rankings based on cost-effectiveness data, respectively. The zero value refers to no relationship between the two variables. The null hypothesis states that the two variables are independent to each other. All analyses were accomplished in Stata version 13.1 by StataCorp LP, Texas, USA.

## Results

### Respondent characteristics

Of the 167 stakeholders who were invited to participate in the interview survey, 93 respondents (55.7%) completed the survey and were included in the analysis—17 from Germany (18.3%), 16 from Hungary (17.2%), 28 from the Netherlands (30.1%), 18 from Spain (19.4%), and 14 from the UK (15.1%). Fifty-eight (62.4%) were males.

Twenty-nine were decision makers (31.2%), 7 were purchasers of services or pharmaceutical products (7.5%), 18 were professionals or service providers (19.4%), 25 were evidence generators (26.9%), and 14 were advocates of health promotion (15.1%). The distributions of stakeholder types across the five countries were not largely different except the UK that had a large proportion of decision makers (approximately 64% compared with the remaining countries that had around 17–33%). The complete compositions of stakeholder types are exhibited in a Additional file 2: Table S1.

### Descriptive statistics of perceived availability and importance

Perceptions on availability of 11 selected interventions are shown in Table 1 (data on all interventions shown in Additional file 2: Table S2). Between the countries, the perceptions on availability varied considerably for almost all of the interventions. The interventions that > 70% stakeholders viewed as ‘available’ in each country were: advertising restrictions/bans, product labelling and information/health warnings on tobacco products, restrictions on sales to minors, restrictions on smoking in workplaces and public places, tax increase, self-help manuals, nicotine replacement therapy, brief physician advice, and group and counselling with or without pharmacotherapies. Stakeholders did not know about the availability of some widely-evaluated interventions such as varenicline (33% of 93 stakeholders), bupropion (29%), computer tailored programs (20.4%), and community pharmacy-based services (17.2%).

**Table 1:** Perceptions on availability of selected interventions across countries

Intervention	Germany (*N* = 17)		Hungary (*N* = 16)		The Netherlands (*N* = 28)		Spain (*N* = 18)		UK (*N* = 14)		Total (*N* = 93)	
Group counselling by specially trained professionals	% A^a^	100	% A	87.5	% A	100	% A	72.2	% A	100	% A	92.5
			% DK^b^	12.5			% DK	0			% DK	2.2
Restrictions on smoking in workplaces and public places	% A	100	% A	100	% A	100	% A	94.4	% A	92.9	% A	97.9
							% DK	0	% DK	7.1	% DK	1.1
Tax increase	% A	100	% A	93.8	% A	89.3	% A	72.2	% A	92.9	% A	89.3
			% DK	6.3	% DK	7.1	% DK	0	% DK	7.1	% DK	4.3
Nicotine replacement therapy	% A	100	% A	100	% A	96.4	% A	88.9	% A	100	% A	96.8
					% DK	3.6	% DK	0			% DK	1.1
Individual counselling by specially trained professionals	% A	100	% A	87.5	% A	92.9	% A	72.2	% A	100	% A	90.3
			% DK	12.5	% DK	7.1	% DK	0			% DK	4.3
Brief advice on smoking cessation given during one GP consultation	% A	100	% A	93.8	% A	100	% A	100	% A	85.7	% A	96.8
			% DK	0					% DK	14.3	% DK	2.2
Telephone counselling	% A	100	% A	68.8	% A	92.9	% A	55.6	% A	85.7	% A	81.7
			% DK	31.3	% DK	7.1	% DK	5.6	% DK	14.3	% DK	10.8
Self-help manuals	% A	100	% A	93.8	% A	92.9	% A	88.9	% A	71.4	%A	90.3
			% DK	6.3	% DK	3.6	% DK	0	% DK	21.4	% DK	5.4
Mobile phone-based interventions	% A	100	% A	43.8	% A	78.6	% A	22.2	% A	64.3	% A	63.4
			% DK	43.8	% DK	17.9	% DK	11.1	% DK	21.4	% DK	18.3
Bupropion	% A	58.8	% A	31.3	% A	64.3	% A	83.3	% A	92.9	% A	65.6
	%DK	41.2	% DK	50.0	% DK	35.7	% DK	5.6	% DK	7.1	% DK	29.0
Varenicline	% A	58.8	% A	43.8	% A	57.1	% A	83.3	% A	92.9	% A	65.6
	% DK	41.2	% DK	56.3	% DK	42.9	% DK	11.1	% DK	7.1	% DK	33.3

^a^A = % of ‘available’

^b^DK = % of ‘don’t know’ and the remaining is % of ‘not available’

**Fig. 1:**
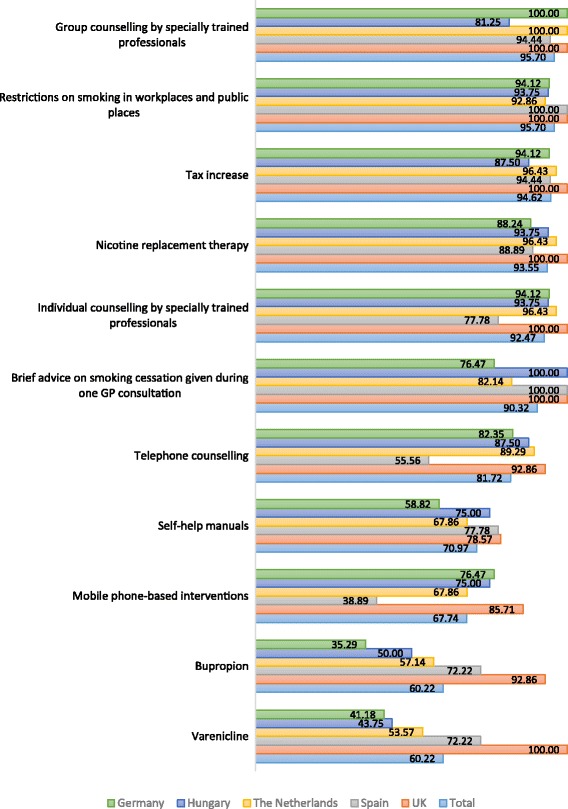
Percentages of selected interventions viewed as somewhat important or important across countries

In each country, a number of interventions were deemed important by > 58% of stakeholders (Table S2). These included nicotine replacement therapy, brief physician advice, the 5-step protocol advice (i.e. ask, advice, assess, assist, and arrange), group and individual counselling by specially trained professionals with and without medications (e.g. NRT or bupropion), advertising restrictions/bans, restrictions on sales to minor, restrictions on smoking in workplaces and public places, and tax increase. Non-conventional therapies were rated important by stakeholders in Germany and Hungary compared to the remaining countries. Non-conventional interventions were viewed to be much less important than the rest of the interventions as only 1.1–6.5% of the whole sample viewed that they were important. Figure 1 shows perceptions on importance of the 11 interventions (somewhat important and important responses combined). The pattern of perceptions was similar when only ‘important’ response was analysed.

### Agreement on perceived availability and importance

Generally, stakeholders tended to agree to a lesser extent on the availability compared with the importance (Table 2). All combined kappa coefficients were statistically significant with *p*-values < 0.001. Stakeholders from Germany, Hungary, and the Netherlands had lower agreement on the availability of interventions than those from Spain and the UK. Slight agreement on availability was observed for every pair of countries except for Spain-UK pair (fair agreement). In general, stakeholders from all countries had slight agreement on the intervention availability (combined kappa = 0.0980; 95% confidence interval = [0.085, 0.111]).

**Table 2:** Agreement on availability and importance of 30 tobacco-control interventions in each country, and between two and five countries

	Combined kappa coefficients*									
In the same country										
	Germany (*N* = 17)		Hungary (*N* = 16)		The Netherlands (*N* = 28)		Spain (*N* = 18)		The UK (*N* = 14)	
	Availability Agreement level	Importance Agreement level	Availability Agreement level	Importance Agreement level	Availability Agreement level	Importance Agreement level	Availability Agreement level	Importance Agreement level	Availability Agreement level	Importance Agreement level
	0.1223	0.2483	0.1826	0.2021	0.1246	0.2711	0.2648	0.2077	0.2293	0.4246
	Slight	Fair	Slight	Slight	Slight	Fair	Fair	Fair	Fair	Moderate
Between the two countries										
	Germany		Hungary		The Netherlands		Spain		The UK	
Germany			0.1318	0.2134	0.1142	0.2509	0.1138	0.2120	0.1021	0.2842
			Slight	Fair	Slight	Fair	Slight	Fair	Slight	Fair
Hungary					0.1198	0.2317	0.1203	0.1961	0.1131	0.2830
					Slight	Fair	Slight	Slight	Slight	Fair
The Netherlands							0.1225	0.2377	0.1057	0.2981
							Slight	Fair	Slight	Fair
Spain									0.2377	0.2739
									Fair	Fair
The UK										
Total (All settings; Number of raters = 93)										
Combined kappa for availability^a^ = 0.0980* (Slight agreement); *p*-value < 0.001; 95% CI = [0.085, 0.111]										
Combined kappa for importance^b^ = 0.2386* (Fair agreement); *p*-value < 0.001; 95% CI = [0.208, 0.253]).										

*All of the combined coefficients were statistically significant with the p-values < 0.001; Kappa and agreement: < 0 = Less than chance agreement, 0.01–0.20 = Slight agreement, 0.21–0.40 = Fair agreement, 0.41–0.60 = Moderate agreement, 0.61–0.80 = Substantial agreement, 0.81–0.99 = Almost perfect agreement. [24]

Within country, variations on importance of interventions included slight to fair agreement (Germany, Hungary, the Netherlands, and Spain) and moderate agreement (UK) (combined kappa in the UK = 0.4246). Every pair of the countries showed fair agreement on importance except Hungary-Spain pair (slight agreement). Stakeholders from all countries had fair agreement on the importance of interventions (combined kappa = 0.2386; 95% CI = [0.208, 0.253]).

### The relationship between availability and importance

Perceived availability of interventions was positively associated with their perceived importance for the following interventions once stakeholder roles, gender and countries were controlled for (*p*-values < 0.05): community pharmacy-based services, computer tailored programs, internet-based interventions, stage-based interventions, and brief advice by a general practitioner and medication (Table 3). The highest correlation was observed for stage-based interventions.

**Table 3:** Relationship between perceived importance and perceived availability

	Descriptive analysis						Ordered logistic regression analysis
Interventions	Available			Not available			Predicted probability of saying important [95% CI]^a^
	Number saying important (%)	Number saying somewhat important (%)	Number saying not important (%)	Number saying important (%)	Number saying somewhat important (%)	Number saying not important (%)	
Community pharmacy-based services (*N* = 78)	30 (50.0)	19 (31.67)	11 (18.33)	6 (33.33)	4 (22.22)	8 (44.44)	54.28%* [38.64–69.93%]
Computer tailored programs (*N* = 75)	25 (41.67)	27 (45.00)	8 (13.33)	3 (20.00)	7 (46.67)	5 (33.33)	44.71%* [30.35–59.08%]
Internet-based interventions (*N* = 77)	35 (53.03)	23 (34.85)	8 (12.12)	1 (9.09)	7 (63.64)	3 (27.27)	53.41%* [39.54–67.29%]
Stage-based interventions (*N* = 70)	34 (59.65)	16 (28.07)	7 (12.28)	5 (38.46)	3 (23.08)	5 (38.46)	72.90%* [57.89–87.91%]
Brief advice by a general practitioner and medication (*N* = 91)	49 (59.76)	27 (32.93)	6 (7.32)	2 (22.22)	2 (22.22)	5 (55.56)	62.53%* [51.24–73.82%]

^a^Predicted probability of saying ‘important’ if the intervention is available, when holding other variables at means. It was estimated by ordered logistic regression model evaluating the effect of availability (yes or no) on importance (not important, somewhat important, or important) controlling for countries, stakeholder roles, and gender

*The regression models were significant with p-values < 0.05. Availability was a significant variable associated with importance for these interventions in the models with the *p*-values of 0.03, 0.004, 0.01, 0.002, and < 0.001, respectively

### Perceived importance score

Table 4 exhibits the scores of all 30 interventions with their categories. The top three interventions were 1) restrictions on smoking in workplaces and public places (score: 1.89; *N* = 92), 2) individual counselling by specially trained professionals with medication (e.g. NRT) (score: 1.86; *N* = 90), and 3) advertising restrictions/bans (score: 1.83; *N* = 92).

**Table 4:** Mean importance score excluding ‘don’t know’ responses

	Intervention (Type of the intervention)	Mean importance score^a^ (SD)	N
1	P^b^: Restrictions on smoking in workplaces and public places	1.89 (0.40)	92
2	C^c^: Individual counselling by specially trained professionals with medication (e.g. NRT or bupropion)	1.86 (0.44)	90
3	P: Advertising restrictions/bans	1.83 (0.46)	92
4	C: Group counselling by specially trained professionals with medication (e.g. NRT or bupropion) (Combined)	1.81 (0.45)	89
5	P: Tax increase	1.80 (0.48)	91
6	B^d^: Individual counselling by specially trained professionals	1.79 (0.49)	89
7	B: Group counselling by specially trained professionals	1.77 (0.49)	92
8	P: Restrictions on sales to minors (Population-level)	1.79 (0.53)	91
9	B: Advice on smoking cessation given according to the 5-step protocol (minimal intervention)	1.76 (0.53)	82
10	M^e^: Nicotine replacement therapy	1.74 (0.53)	91
11	B: Brief advice on smoking cessation given during one general-practitioner consultation	1.70 (0.64)	93
12	M: Varenicline	1.59 (0.64)	61
13	P: Mass media campaigns	1.53 (0.69)	89
14	C: Brief advice by a general practitioner and medication	1.44 (0.70)	91
15	P: Product labelling and information/ Health warnings on tobacco products	1.47 (0.75)	91
16	B: Telephone counselling	1.36 (0.67)	85
17	M: Bupropion	1.40 (0.72)	65
18	B: Stage-based interventions	1.39 (0.77)	70
19	B: Internet based interventions	1.32 (0.72)	77
20	B: Computer tailored programs	1.20 (0.72)	75
21	B: Mobile phone-based interventions	1.14 (0.69)	76
22	B: Community pharmacy-based services	1.22 (0.82)	78
23	B: Self-help manuals	1.08 (0.78)	90
24	N^f^: Hypnosis-based interventions	0.40 (0.59)	75
25	N: Acupuncture	0.41 (0.63)	81
26	N: Aromatherapy	0.15 (0.40)	72
27	N: Magnetic resonance therapy	0.12 (0.37)	68
28	N: Homeopathy	0.28 (0.53)	78
29	N: Smokeless tobacco	0.30 (0.59)	73
30	N: Herbs	0.22 (0.56)	74

^**a**^The score ranged from 0 to 2 with 0 representing not important and 2 representing important. The ‘don’t know’ response was missing

^b^P = Population-level intervention

^c^C = Combined pharmacological and behavioural intervention

^d^B = Behavioural intervention

^e^M = Pharmacological intervention

^f^*N* = Non-conventional intervention

### Comparison of importance of intervention

Table 5 puts the stakeholders’ perceived importance scores into perspective by comparing their rank order with that by published cost-effectiveness evidence. A total of 10 interventions were included for this comparison. The top three interventions by the perceived importance score were (1) individual counselling by professionals with medication (score: 1.86); (2) group counselling by professionals with medication (score: 1.81); and (3) nicotine replacement therapy (score: 1.74). These first three interventions would also be most cost-effective based on the published review. The large differences in importance of interventions between stakeholder views and cost-effectiveness evidence were apparent for self-help manuals, telephone counselling, community pharmacy-based services and mass media campaigns. These interventions ranked lower by importance score than by cost-effectiveness criteria. Brief advice by a general practitioner ranked marginally higher by importance score than by cost-effectiveness criteria. Kendall rank correlation coefficient was 0.40 with the *p*-value of 0.11, indicating that ranking based on the importance score did not statistically agree with the ranking based on cost-effectiveness evidence (95% CI = [− 0.09, 0.89]).

**Table 5:** A head-to-head comparison of selected tobacco-control interventions when ranked by stakeholders’ views and existing cost-effectiveness evidence

Intervention	Mean importance score^a^	Rank by mean importance score	Rank by cost-effectiveness^b^	Median cost/QALY (£)^b^	Comparator^b^	Range^b^
Individual counselling by specially trained professionals with medication (e.g. NRT or bupropion)	1.86	1	1	Dominates^c^	Background quit rate	NA
Group counselling by specially trained professionals with medication (e.g. NRT or bupropion)	1.81	2	1	Dominates^c^	Background quit rate	NA
Nicotine replacement therapy	1.74	3	1	Dominates^c^	Background quit rate (no intervention)	NA
Brief advice on smoking cessation given during one general-practitioner consultation	1.70	4	6	732	Background quit rate	577–1677
Mass media campaigns	1.53	5	2	49	Background quit rate	NA
Brief advice by a general practitioner and medication	1.44	6	7	2110	Background quit rate	1664–4833
Stage-based interventions	1.39	7	8	3033	No intervention (aggregate of controls)	NA
Telephone counselling	1.36	8	4	427	Usual care or intervention but no telephone counselling	139–1602
Community pharmacy-based services	1.22	9	5	546	Usual care	438–655
Self-help manuals with brief advice (5 min)	1.08	10	3	370^b^	Background quit rate	292–847
Kendall rank correlation coefficient evaluating the association between the two rankings = 0.40; *p*-value = 0.11; 95% CI = [−0.09, 0.89])						

^a^Calculated using stakeholders’ responses on a 3-point Likert scale, ^b^Sourced from Owen et al. (2011), ^c^Implies intervention is less costly with more benefit

## Discussion

To the best of our knowledge, this study is the first of its kind to improve our understanding of stakeholder views around availability and importance of interventions with respect to cross-context transferability of economic evidence on tobacco control. It appears that stakeholders or end-users of research generally have considerably different opinions on the availability of the interventions but tend to agree with each other within and across countries more on the importance of the interventions. There was positive relationship between availability and importance of some interventions. The stakeholder perception on the importance of interventions did not necessarily match what existing cost-effectiveness evidence suggests.

Our findings challenge the commonly-held notion that widely evaluated tobacco control interventions (e.g. bupropion, varenicline, or community pharmacy based interventions) are already known in the new settings. Bupropion and varenicline were available in all of the countries (Germany, Hungary, the Netherlands, Spain, and the UK) according to WHO [28] but our findings show that 36–56% of stakeholders in Germany, Hungary, and the Netherlands and 6–11% in Spain and the UK did not know that. Therefore, increasing awareness of these interventions should be recommended. Also, the assumption that ‘stakeholders know about the interventions’ must be checked prior to facilitating any evidence transfer to a new context/setting.

The significant positive relationship observed between what stakeholders viewed as ‘available’ and ‘important’ for some interventions implies that decisions about implementing a new intervention, or making it available, may essentially depend on the extent to which those interventions are viewed as important.

Moreover, the difference in rankings by stakeholders’ perceptions on importance and cost-effectiveness data provides some hints that there may be other factors behind how interventions are valued in a country. Burchett et al. (2013) found that one of the factors affecting stakeholders’ perceptions was their previous experiences and beliefs about the interventions [29]. Thus, recognising this difference prior to any evidence transfer work is critical as the interventions that are perceived ‘important’ by stakeholders are also the ones that may be deemed applicable to new settings [17, 18].

Some study limitations should be recognized. First, the availability question in the survey did not clearly specify whether availability referred to the product being available on the market in the respective health care system or available under coverage by social security (i.e., as part of the National Health Service or statutory sickness funds, etc.). This might have caused some different interpretations by respondents. If this was the case, this might have caused larger heterogeneity in the responses about the availability of, particularly, pharmaceutical products compared to general-practitioner advice or public health campaigns.

The second limitation was the sample with the response rate of approximately 56%. Although our sample had in general similar numbers of respondents in each country, every country had different stakeholder composition. Majority of the respondents were health advocates in Germany, evidence generators in Hungary and the Netherlands, and decision makers in Spain and the UK. This may explain why the agreement on the availability in Spain and the UK were higher than that in other countries. Decision makers are more likely to be familiar with intervention availability and may have more opportunities to discuss this with each other than other stakeholder groups. Most non-respondents were decision makers from the Netherlands but there were still a good number of them participating (eight while three to nine from the remaining countries). It is important to realise this caveat before generalising the study results.

The study results may be helpful to inform the selection of cost-effective tobacco-control interventions that are transferable. For example, non-conventional interventions which are viewed as less important in every country compared with other interventions may be excluded from an evidence transfer project. Besides, the interventions which were widely available and considered valuable such as nicotine replacement therapy, counselling-related interventions, and population-level interventions may be included. Being able to transfer economic evidence, instead of fully conducting context-specific research, will lead to gaining substantial savings in research resources in other countries. This is important to countries such as Central and Eastern Europe, where potential to save lives from tobacco control is enormous but the resources to conduct context-specific economic evaluations of interventions are extremely limited. Therefore, strong engagement with key stakeholders, focussing on better communication, has potential to improve evidence transferability.

Although different from many transferability studies [9] where the focus appears to gather local input parameters to the economic model being transferred, this study adds to transferability pathway by understanding first whether stakeholders within and across countries have similar views on the economic evidence being transferred and the extent of the difference when their views vary. This understanding informed selection of interventions in the European study (EQUIPT) [16] and thus proved to be vital to improve transferability of evidence.

## Conclusions

European stakeholders show low agreement on the availability and fair agreement on the importance of tobacco-control interventions within and across countries. These intrinsic differences in stakeholder views must be addressed while transferring economic evidence Europe-wide. Strong engagement with key stakeholders, focussing on better communication, has a potential to mitigate this challenge, and save scarce research resources.

## Additional files

Additional file 1: Interview Guide and Questionnaires of the EQUIPT Stakeholder Survey. (DOCX 86 kb)

Additional file 2: **Table S1.** Distribution of stakeholders in each country. **Table S2.** Perceived availability and importance of tobacco-control interventions across countries. (DOC 278 kb)

## Abbreviations

EQUIPT: European-study on Quantifying Utility of Investment in Protection from Tobacco
EU: European Union
METC: Medisch-ethische toetsingscommissie
NICE: The National Institute for Health and Care Excellence
QALYs: Quality Adjusted Life Years
ROI: Return on Investment
UK: United Kingdom
