# Supplementary material for: Understanding perceived availability and importance of tobacco control interventions to inform European adoption of a UK economic model: a cross-sectional study
Source: BMC Health Serv Res. 2018 Feb 14;18:115. doi: 10.1186/s12913-018-2923-2 (PMC5813331; doi:10.1186/s12913-018-2923-2)
Supplement: Supplementary file 1 — Interview Guide and Questionnaires of the EQUIPT Stakeholder Survey. (DOCX 86 kb) [file 12913_2018_2923_MOESM1_ESM.docx]

**EQUIPT Work Package 1 Task 3: Stakeholder Survey**

**Interviewer’s Manual and Questionnaire**

1. **Introduction**

The Stakeholder Survey forms a part of WP1 and is designed to collect views of stakeholders^[[1]](#footnote-1)^ about their needs for and their intention to use economic tools, such as the Tobacco Return on Investment (ROI) Tool, to support their activities such as making purchase/reimbursement decisions, implementing services and making the business case for tobacco control.

The Project will interview 15-20 stakeholders in each sample country (i.e. Germany, Hungary, Spain, the Netherlands and the UK). These respondents will be asked questions about their professional roles and a number of questions that captures their needs for and the intention to use the ROI tools. To facilitate their understanding about the tool, a video will be shown as a part of the interview.

As a country interviewer, your task is to conduct this interview in the standardised way. This is why this manual provides you with the information that is expected to be understood by all country interviewers in the same way.

If you are not sure about anything about the survey process and/or the questionnaire, please contact the Task Lead (Zoltán Vokó, zoltan voko@syreon.eu) immediately.

1. **Stakeholders – definitions**

For the purpose of this project, a stakeholder is defined as an individual (or an organisation) who has an interest in and/or contributes to tobacco control agenda in their professional roles.

The Project has identified five types of stakeholders:

a. Decision makers

b. Purchaser of services/pharma products

c. Professionals/service providers

d. Evidence generators

e. Advocate of health promotions

f. Any other role not mentioned above

1. **Sample**

Each country is expected to interview 15-20 stakeholders from the above six categories. It is required that at least 2 stakeholders from category ‘a’ to ‘e’ are included in the final sample. The category ‘f’ is to allow for any stakeholders who cannot be categorised as above.

Note that, in order to have a final sample of 15-20 stakeholders, you will need to approach about 5 stakeholders in each category.

Attach an ID number to each stakeholder. Keep this record in a password protected file and after the completion of the interviews send it to the country leads.

1. **Organising the survey**

You will need to work with your Country Lead to discuss and finalise the list of stakeholders that you would like to approach. The ENSP (Cornel Radu-Loghin) may have a database of potential stakeholders and therefore it is recommended that they should be approached to suggest names for your country.

Once the list is created, please send it to the Task Lead for their record. You will then need to approach the stakeholders with the following (all will be provided to you):

1. Invitation letter
2. Information Sheet
3. Additional documents

You will need to keep a diary when you approached the stakeholder and when and where the interview is agreed to take place. If you have not received any response, you will need to send reminders as agreed in the protocol.

On the day of the interview, please make sure that you arrive at least 15 minutes earlier and present yourself as relaxed, confident and charming colleague who want to explore their (the stakeholder’s) views about the ROI tools. Never give any impression to the stakeholder that you are there to enquire about their daily decision making activities – this is NOT the purpose of the interview and this should strictly be avoided. Make the interview as pleasant experience as possible for both of you.

Before you go for the interview, fill in the front page of the questionnaire including the ID number of the stakeholder as well. Indicate the same number on the interviewee version of the questionnaire, too.

1. **Interviewing stakeholders**

Note that interviewing should not be regarded as a mechanical process, rather it is an art. Being relaxed, confident and helpful makes the atmosphere pleasant and you are likely to obtain rich data you are after. Spend the first one or two minutes to build rapport, by talking about anything that is not related to the interview. For example, you could talk about the weather, your journey, how wonderful location their office is in, etc. This helps you both ‘break the wall’ and get in to a natural flow of conversation.

Once you feel that both of you are at ease, start the interview. You will need to obtain their informed consent. Hand over the form and ask them to kindly sign it. If needed, explain what each of the statements in the form mean. Highlight the fact that:

1. Their participation is entirely voluntary and they can withdraw at any time
2. That their views will be kept confidential and all data are anonymised

After the introduction start the tape recorder and record the ID number on the tape and the whole interview. Ask the interviewees for permission of the recording.

At question 8 hand over the interviewee version of the questionnaire and ask the interviewees to mark their answers on their copies. That version of the questionnaire does not contain question marks as potential answers. If stakeholders are not willing to answer a question, ask them whether they feel neutral (i.e. ‘4’) and otherwise: leave it open (i.e. the question mark).

It is not uncommon that you find yourself answering some questions from the stakeholders. This situation arises when the stakeholder does not understand any question or terms used in the questionnaire. The purpose of this guide is to equip you to handle such a situation. Therefore, it is vital you read this guide carefully. Do not assume any answer- if you don’t know how to answer any question, be frank about it.

Unless the stakeholder wants his team to be present in the interview, make sure that you interview him/her alone. This is to assure confidentiality.

It is vital that you should be neutral throughout the interview. Your role is to hear the stakeholders’ answer and not to provide them with the suggestions. Don’t show that you are agreeing or disagreeing to their answers; rather encourage them to complete the question with their own answer.

As a part of the interview, you will need to show a video to the stakeholder. During this show if the stakeholder wants to ask you a question, pause the video and answer their questions. Before you start the video again, make sure there is no further question from them at this stage.

If any stakeholder gives an ambiguous answer, ask further questions, e.g. “please can you explain a little bit more what you exactly mean here?” or you can even say, “You can take a bit more time to answer this question if you want”.

You may find that some stakeholders are hesitant to answer a particular question(s). In this case, you will need to encourage them. The best way to do this is to talk a little bit more about the question, e.g. explain it further. However, sometime people are hesitant to answer a question because they feel bored. In this case, talking about something totally unrelated (e.g. weather, skiing, etc.) may re-interest them.

Finally, do not rush the interview. Ask questions slowly and make sure that it is answered. Having a little pause between two questions will help both of you to get ready for the next question. If you think the stakeholder is answering your question without thinking, encourage them to consider the answers carefully by allowing some more time. As you go along, please make a note that all questions have indeed been answered (completeness check).

Once the interview is complete, thank them for their time and input. Assure them once again that their inputs are important for this project and all data will be kept confidential. Assure them that once the analysis is done, the anonymised results will be communicated via the Project website. Ask them if they wish to receive results via email instead and check whether the email that you hold is the correct one.

1. **Language of the interview**

The interview will be held in the country language. All questionnaire and supporting materials are translated in the country language.

1. **Post-interview**

Email the stakeholder, thanking them for their time and inputs (a standard text will be provided).

Check completeness of the data again, fill in additional information including the key words of the free text answers recorded, and any other (e.g. through your notes) if the need be, scan the questionnaires and save it as password encrypted PDF. Save the tape recorded original files, as well. Do not forget that two questionnaires belong to each interview, one which is filled in by the interviewer and another which is filled in by the interviewee. Scan both! Send the PDF to Kei Long Cheung (kl.cheung@maastrichtuniversity.nl).

The original copy should be put in a sealed envelope and kept by the country lead.

It is your responsibility to maintain confidentiality of all data.

1. **Questionnaire guide**

**Interview Guide - Page 1**

This section has following details which you will need to fill in before the interview:

1. Country – state the country where the interview is taking place
2. ID Number – identification number for the stakeholder (contact Kei Long for the list)
3. Date of interview – write the date on which the interview is taking place
4. Role of the interviewee – tick one from the list provided
5. Name of the interviewer- write your name
6. Duration of the interview: record the starting date (DD:MM) and time(HH:MM) and the end time (HH:MM) of the interview, here.

You should then sign this page.

**Interview Guide - Part A**

The objective of the first section of this part is to introduce you to the stakeholder. You should practice the text before the interview so that you can introduce yourself without having to read from this section. Make sure you have as much eye contact as possible and your introduction is natural.

Before you move onto the next question (Q7), ask their permission whether it is OK to proceed and record the interview and whether they have any question at this stage. Start the tape recorder.

- **Q7**

The objective of this question is to obtain more data about the type of the stakeholder you are interviewing. You should be collecting more information about their roles and responsibility. Please, take a note in the space provided as the stakeholder talks (and additional if needed based on the record). Make sure, however, that you maintain as many eye contacts as possible in between.

- **Q8**

Provide the information summarized in the framed text before the question. Hand over the interviewee version of the questionnaire and explain the interviewees that you will be asking them to mark on a 7-item scale what extent they agree with some statements, 1 meaning “strongly disagree”, 7 meaning “strongly agree”, 4 meaning being neutral, not having an opinion on a statement.

The objective of this question is to collect the stakeholder intention to use an economic tool to support their roles/jobs on the basis of their current understanding. Therefore, they may ask you a question that they don’t know enough about the economic tool and hence do not want to answer the question. If this happens, you should say that you will be showing a video in a minute but just wanted to have their rating based on their current understanding. Also, mention that you will ask the same question again towards the end of the interview when they would have explored the ROI tool a bit further. Note that this question is asked twice to measure any change in intention to use; therefore it is important that you obtain the response from the stakeholder on this question.

- **Q9**

In this question, we are measuring the level of knowledge stakeholders have regarding HTA information. Therefore any questions or confusion should be responded by: ‘’In this question we are interested how much you know about certain information. Please circle a number that you feel represents best the correct answer.’’

- **Q10**

In this question, we are measuring the level of risk perception regarding smoking and the use of HTA. ‘’It is important that you indicate on this scale you feel best about in answering the question.’’ ‘’However, concerning time issues we need to proceed efficiently and we ask you to fill in the scales that generally represent your feelings best.’’

- - F. Effect meaning efficacy.

Show the video. Pause, if the interviewees ask questions.

**Interview Guide - Part B**

- **Q11**

Follow the instruction about scales given at Q8.

If the respondent asks for clarification about the following terms, then give her/him based on these definitions:

- *Prevalence*: the proportion of persons in a population with a certain characteristics or with a history of an event at a particular time. For example, prevalence of diabetes in 2010 means what proportion of the population had diabetes in the year 2010.
- *Budget Impact:* the total cost of the introduction of a smoking cessation intervention that incurs at the level of the organisation
- **Q12**

If the answer is “NO”, then do not read the second question (“Could you please…”) but go to question 13.

**Interview Guide - Part C**

1. This part of the questionnaire will be used to investigate stakeholders’ perceptions of the Tobacco ROI Tool. It consists of mainly quantitative questions, but also some open-ended items. Using the double questionnaire method, it is important to instruct the interviewee that the scale-type answers are those which the interviewees should mark themselves. Interviewers need to watch that all these scales are marked. They should avoid copying these into the questionnaire they are filling in during the interview, because it will result in errors. The interviewers should fill in keywords for the open-ended questions on the spot and based on the record of the interview and instruct the interviewee that it is unnecessary to fill in the open-ended items.
2. This part of the questionnaire investigates the motivational factors and the intention state of the adoption of the ROI tool (I-Change). Following topics are therefore addressed: attitude, social influences, self-efficacy, and intention to adopt.
3. Answer any questions from the respondent frankly. Interviewees may ask questions or want to talk further about the topics you bring up during the interview. It is important not to interrupt the flow of the interview concerning time issues. Tell the interviewee that there is not much time and we have to proceed and be as efficient as possible: ‘’Many topics within this interview are very interesting, but concerning time issues we have to proceed as efficient as possible.’’
4. If the interviewee gives an ambiguous answer in open-ended questions, try to probe in a neutral way, asking questions such as: ‘’Can you explain a little more?”
5. If the interviewee gives an ambiguous answer in quantitative questions, try to force an answer by telling the participant that it is important that he or she indicates on the Likert scale what he or she generally feels it is best to answer. Some interviewees may indicate that he or she is not able to provide a number as the answer depends on the situation. Some interviewees may respond with lengthy explanations why it is difficult to answer. However, it is important that the interviewee use the Likert scale to answer the items. Following examples may be used in these situations: ‘’It is important that you indicate on this scale you feel best about in answering the question.’’ ‘’It is indeed interesting to elaborate on these questions and it may be hard to answer. However, concerning time issues we need to proceed efficiently and we ask you to fill in the scales that generally represent your feelings best.’’
6. If the interviewee is not willing to answer quantitative questions, ask them whether they feel neutral about the statement: ‘’As you are not able to provide an answer; does this mean that you feel neutral about this statement?’’ A ‘4’ should be marked by the interviewer if the answer is positive. If this is not the case, the interviewer should mark the question mark ‘?’.
7. The wording of the questions and their sequence in the questionnaire must be maintained. If the interviewee has not understood the question, you should repeat the question slowly and clearly. If there is still a problem, you may reword the question, being careful not to alter the meaning of the original question. Provide only the minimum information required to get an appropriate response.

- **Q13**

This question includes items measuring the attitude of the interviewee regarding the Tobacco ROI tool. Pros and cons are investigated by asking about the perceived advantages and disadvantages. Read the question - Could you please indicate on a scale from 1 to 7 - 1 meaning “disagree” and 7 meaning “agree” - to what extent you agree with the following statements? Please circle your answer in the column to the right - and tell the respondent: ‘’I will now read you some statements, please circle the answer after each statement on the right.’’ ‘’Items are about the advantages and disadvantages that you expect the ROI tool to have.’’

Attitude

- - A. Asking the interviewee before statements about advantages and disadvantages may result into a general feeling whether the interviewee feels that there are more pros than cons.
  - B-L. These items measure the perceived advantages. Tell the respondent: ‘’ What could be **advantages** for you of the Tobacco ROI tool?’’ Within a second, proceed: ‘’A tool such as the Tobacco ROI tool: ’’ Proceed to the next item after the interviewee circled their answer on the Likert scale.
    - *decision making* refers to making decisions concerning investments in tobacco control strategies.
    - smoking cessation methods refers to smoking cessation interventions or tobacco control measures.
    - *service delivery* refers to the delivery of outputs of their work.
    - *Interventions in item K* refers to smoking cessation interventions.
  - M. This is an open-ended question exploring whether there are more perceived advantages not asked in the scale. If the interviewee does not understand the question, reword it: ‘’Based on the video earlier, what other **advantages** do you expect the tool to have for you?’’ Write the advantages in keywords, for example: ‘’looking fancy.’’
  - N-V. These items measure the perceived advantages. Tell the respondent: ‘’ What could be **disadvantages** for you of the Tobacco ROI tool?’’ Within a second, proceed: ‘’A tool such as the Tobacco ROI tool:…………’’ Proceed to the next item after the interviewee circled their answer on the Likert scale.
    - Item Q, is not useful (for your work or decision making)
    - *Data input* refers to the actions that the user has to take when working with the tool.
  - W. This is an open-ended question exploring whether there are more perceived disadvantages not asked in the scale. If the interviewee does not understand the question, reword it: ‘’Based on the video earlier, what other **disadvantages** do you expect the tool to have for you?’’ Write the advantages in keywords, for example: ‘’looking old-fashioned.’’
- **Q14**

This question includes items measuring social influences of the interviewee (A-I), self-efficacy (J-U), and the intention to adopt the ROI tool (V-AA). Read the question - Could you please indicate on a scale from 1 to 7 - 1 meaning “disagree” and 7 meaning “agree” - to what extent you agree with the following statements? Please circle your answer in the column to the right - and tell the respondent: ‘’I will now read you some statements, please circle the answer after each statement on the right.’’

Social influences

- - A-F. These items measure the social support for adopting the ROI tool. We are interested in the perception of the support that they encounter from others in adopting the ROI tool.
    - *Boss* refers to the supervisor in the interviewee’s job.
    - *Support* means to provide for or maintain, by supplying with money or necessities. Support here also means to argue in favor of the adoption of the ROI tool.
    - *decision making* refers to making decisions concerning investments in tobacco control strategies.
  - G. This is an open-ended question exploring the perceived support for adopting the ROI tool. If the interviewee does not understand the question, reword it: ‘’Based on the video earlier, are there people – not mentioned earlier – that will probably support you in adopting the ROI tool?’’ Write the answer in keywords, for example: ‘’my partner.’’
  - H. This question addresses the interviewee’s perception of social resistance in adopting the ROI tool. If the interviewee does not understand ‘not support’, reword the question: ‘’ I would encounter resistance using the Tobacco ROI tool.’’
  - I. This is an open-ended question exploring the perceived resistance for adopting the ROI tool. If the interviewee does not understand the question, reword it: ‘’Based on the video earlier, are there people – not mentioned earlier – that will probably be against you in adopting the ROI tool?’’ Write the answer in keywords, for example: ‘’my partner.’’

Self-efficacy

- - J-T. Interested in self-efficacy, we quantify the interviewee’s perception of his or her capability to adopt (use) the ROI tool. Tell the respondent: ‘’How confident are you about using the Tobacco ROI tool?’’ Within a second, proceed: ‘I am confident that:…………’’ Proceed to the next item after the interviewee circled their answer on the Likert scale.
  - *Persons as myself* refers to you and colleagues with similar background and work.
  - *Outputs of the tool* are results coming out from the ROI tool when using it.
- U. This is an open-ended question exploring the perceived difficulties for adopting the ROI tool. Write the answer in keywords, for example: ‘’need training.’’
- **Q15**

This question includes items measuring the intention of the interviewee to adopt the Tobacco ROI tool.

- A-E is related to the intention to adopt the Tobacco ROI tool. The rationale of the sequence of items is that the behavior increases in intensity. Z is concerned about the interviewee’s need to search for more information to translate their intention into deeds.
  - The time (e.g. the next month, the next year) refers to the usage of the ROI tool regularly within this time horizon.

**Interview Guide Part D**

The objective of this part of the questionnaire is to assess the availability of tobacco control measures & smoking cessation interventions

- **Q16**

Ask the stakeholder to circle appropriate box(es). The table lists potential interventions and they should indicate whether the listed intervention is available in their country, and rate whether the listed intervention is important to them (scale 1-3) and whether they are available in their locality/country (Y/N).

- - Herbs meaning natural herbs, like peppermint.
- **Q17**

This question is an open-ended question. The objective is to collect stakeholders’ expectation on participating in the EQUIPT project and how their organizations may benefit from the project.

- **Q18**

The objective here is to collect stakeholder views on smoking attributable diseases that they are interested in. In the blank space, list all diseases they mention.

- **Q19**

This space is for you to comment on how the interview went and whether there are any lessons to be learnt for the next one.

 

The first page is to be filled in before the interview.

1. Country: …………………………………………..
2. ID number: ………………………………………………………….
3. Date of the interview (dd-mm-yy): ………………………………………………………………
4. Role of the interviewee:
   1. Decision maker
   2. Purchaser of services/pharma products
   3. Professional, service provider
   4. Evidence generator
   5. Advocate of health promotion
   6. Other, indicate:
5. Name of the interviewer (in capitals): ……………………………………………………………………………….
6. Duration of the interview: ……………………………………………………………………………….

I hereby declare, that I handle the information collected securely, and provide it only for authorised person participating in the EQUIPT project. The data is anonymised before use.

………………………………………….

Signature of the interviewer

**Part A. Introduction**

Hello, my name is ……….. from ………… We are participating in an international research project that investigates how the Tobacco Return On Investment Tool, developed by the National Institute for Health and Care Excellence (NICE) in the UK, can be adapted and transferred to different European countries, including [your country]. Hereafter, this tool is referred to as the “Tobacco ROI tool” for convenience.

The specific aims of the project are:

- to develop Tobacco ROI tools in four EU countries (Germany, Hungary, the Netherlands and Spain);
- to assess the needs for and ideas of using the Tobacco Return On Investment Tool to compare the results of ROI analysis across these countries;
- to transfer policy recommendations to other European countries not included in the sample.

In Part I. of the interview I will ask some general questions about setting priorities (what kind of information do you use when you make decisions on investments in tackling the scourge of tobacco?) related to tobacco control measures and smoking cessation interventions. In Part II. I will show you a presentation of the Tobacco ROI tool, and then I will ask you some questions about this tool.

We will tell you more about the Tobacco ROI tool in a minute.

To facilitate the best use of the ROI tool, we would ask for further input from you to fine tune the presentation of the tool and its results to ensure its relevance for practice.

Thank you for agreeing to provide a perspective. We appreciate your advice on how we can best shape this work so that it can provide greatest practical value to you. Our conversation should last approximately 40 minutes. I really appreciate your time and inputs to this process.

May we start the interview now? If you do not mind, I would tape-record it to save time. Is that okay?

Start the tape recorder and record the ID number

1. Can you briefly introduce yourself, your role and your responsibilities?

___________________________________________________________________________________________________________________________________________________________________________________________________________________________________________________________________________________________________________________________________________________________________________

The Tobacco ROI tool basically calculates locally the return on investment for different packages of tobacco control measures. Please answer the upcoming question based on the information provided earlier. Here is your own copy of the questionnaire. Please answer the upcoming question based on the information provided earlier. We will provide you with more information about the tool in a minute

1. Could you please indicate on a scale from 1 to 7, 1 meaning “strongly disagree” and 7 meaning “strongly agree” to what extent you agree with the following statement: ‘’I have the intention to use an economic tool such as the Tobacco ROI for decision making/information gathering concerning tobacco control.’’ Please circle your answer as appropriate in the questionnaire.

| Strongly Disagree | |  |  | Strongly Agree | | |  |
| --- | --- | --- | --- | --- | --- | --- | --- |
| 1 | 2 | 3 | 4 | 5 | 6 | 7 | ? |

1. Could you please indicate on a scale from 1 to 7 - 1 meaning “strongly disagree” and 7 meaning “strongly agree” to what extent you agree with the following statements? Please circle your answer in the column to the right.

|  | Strongly Disagree | | | | Strongly  Agree | | | |  |
| --- | --- | --- | --- | --- | --- | --- | --- | --- | --- |
| 1. “Incremental costs” means how much the studied intervention itself costs more or less than the comparator intervention | 1 | 2 | 3 | 4 | | 5 | 6 | 7 | ? |
| 1. When interventions in itself are cheap, they are always cost-effective compared to another intervention | 1 | 2 | 3 | 4 | | 5 | 6 | 7 | ? |
| 1. “Willingness to pay” means how much a society is willing to pay for a quality adjusted life year or QALY | 1 | 2 | 3 | 4 | | 5 | 6 | 7 | ? |
| 1. My intervention can be cost-effective compared to another intervention, even when its societal costs are higher than the regular care | 1 | 2 | 3 | 4 | | 5 | 6 | 7 | ? |
| 1. In a health care payer perspective, indirect costs in full (such as productivity losses) are included | 1 | 2 | 3 | 4 | | 5 | 6 | 7 | ? |

1. Could you please indicate on a scale from 1 to 7 - 1 meaning “strongly disagree” and 7 meaning “strongly agree” to what extent you agree with the following statements? Please circle your answer in the column to the right.

|  | Strongly Disagree | | | | Strongly  Agree | | | |  |
| --- | --- | --- | --- | --- | --- | --- | --- | --- | --- |
| 1. The smoking epidemic is not severe in my country | 1 | 2 | 3 | 4 | | 5 | 6 | 7 | ? |
| 1. Most smoking cessation interventions are effective | 1 | 2 | 3 | 4 | | 5 | 6 | 7 | ? |
| 1. Most smoking cessation interventions are cost-effective | 1 | 2 | 3 | 4 | | 5 | 6 | 7 | ? |
| 1. It is important to use smoking cessation interventions because smoking kills a lot of people | 1 | 2 | 3 | 4 | | 5 | 6 | 7 | ? |
| 1. It is important to use smoking cessation interventions because smoking costs a lot for the society | 1 | 2 | 3 | 4 | | 5 | 6 | 7 | ? |
| 1. It is unacceptable that we use smoking cessation interventions without knowing their efficacy | 1 | 2 | 3 | 4 | | 5 | 6 | 7 | ? |
| 1. It is unacceptable that we use smoking cessation interventions without knowing their cost-effectiveness | 1 | 2 | 3 | 4 | | 5 | 6 | 7 | ? |

**Part B. Needs assessment**

Now, let me show you a short video about the return on investment tool.

Show the video

1. Could you please indicate on a scale from 1 to 7 - 1 meaning “strongly disagree” and 7 meaning “strongly agree” to what extent you agree with the following statements? Please circle your answer in the column to the right.

| If I were to use a tool such as the Tobacco ROI tool, it needs to give me: | Strongly Disagree | | | | Strongly Agree | | | |  |
| --- | --- | --- | --- | --- | --- | --- | --- | --- | --- |
| 1. Data on **prevalence** of smoking as reported in surveys and scientific studies | 1 | 2 | 3 | 4 | | 5 | 6 | 7 | ? |
| 1. Data on **costs of smoking** | 1 | 2 | 3 | 4 | | 5 | 6 | 7 | ? |
| 1. Data on **quality of life** as reported in surveys and scientific studies | 1 | 2 | 3 | 4 | | 5 | 6 | 7 | ? |
| 1. Data on **mortality** due to smoking | 1 | 2 | 3 | 4 | | 5 | 6 | 7 | ? |
| 1. **Effectiveness** of smoking cessation interventions (such as quit and relapse rate) as reported in scientific studies | 1 | 2 | 3 | 4 | | 5 | 6 | 7 | ? |
| 1. **Cost-effectiveness** data comparing the cost of smoking cessation interventions with its health and wider benefits | 1 | 2 | 3 | 4 | | 5 | 6 | 7 | ? |
| 1. **Budget impact** reflecting financial outcomes specifically at organisational level | 1 | 2 | 3 | 4 | | 5 | 6 | 7 | ? |
| 1. Are there any other types of evidence that you consider important about the decision/information gathering on the implementation of tobacco control measures and smoking cessation interventions?   ___________________________________________________________________________________________________________________________________________________________________________________________________________________________________________________________________________ | | | | | | | | |  |

1. Do you know about any other economic/financial models/tools of tobacco control measures/smoking cessation interventions (such as a decision tree or the WHO tobacco tax simulation model)?
   1. No → Go to question 12
   2. Yes

Could you please give one or more examples?

______________________________________________________________________________________________________________________________________________________________________________________

**Part C. Tobacco ROI Tool**

Now we would like to ask you some questions about the Tobacco ROI Tool just presented to you.
We are interested in your expectations or beliefs about the upcoming tool

1. Could you please indicate on a scale from 1 to 7 - 1 meaning “strongly disagree” and 7 meaning “strongly agree” to what extent you agree with the following statements? Please circle your answer in the column to the right.

|  | Strongly Disagree | | | | Strongly  Agree | | | |  |
| --- | --- | --- | --- | --- | --- | --- | --- | --- | --- |
| 1. The Tobacco ROI tool has more advantages than disadvantages for me | 1 | 2 | 3 | 4 | | 5 | 6 | 7 | ? |
| ***What could be advantages for you of the Tobacco ROI tool?  I belief that a tool such as the Tobacco ROI tool:*** |  |  |  |  | |  |  |  |  |
| 1. provides me with financial justification for my decision making/ information gathering | 1 | 2 | 3 | 4 | | 5 | 6 | 7 | ? |
| 1. is easy to use | 1 | 2 | 3 | 4 | | 5 | 6 | 7 | ? |
| 1. provides me with up-to-date information | 1 | 2 | 3 | 4 | | 5 | 6 | 7 | ? |
| 1. provides relevant outcomes for my organisation | 1 | 2 | 3 | 4 | | 5 | 6 | 7 | ? |
| 1. provides sufficient scientific support for decision making | 1 | 2 | 3 | 4 | | 5 | 6 | 7 | ? |
| 1. provides added value to the information currently used | 1 | 2 | 3 | 4 | | 5 | 6 | 7 | ? |
| 1. helps me decide which smoking cessation methods I should choose | 1 | 2 | 3 | 4 | | 5 | 6 | 7 | ? |
| 1. integrates all relevant information available to calculate cost-effectiveness | 1 | 2 | 3 | 4 | | 5 | 6 | 7 | ? |
| 1. can calculate the cost-effectiveness outcomes over different periods such as 2 years, 5 years, 10 years, and lifetime | 1 | 2 | 3 | 4 | | 5 | 6 | 7 | ? |
| 1. can assess cost-effectiveness of potential new interventions | 1 | 2 | 3 | 4 | | 5 | 6 | 7 | ? |
| 1. helps increase efficiency in service delivery | 1 | 2 | 3 | 4 | | 5 | 6 | 7 | ? |
| 1. What other advantages would a tool such as the Tobacco ROI tool have for you?   ____________________________________________________________________________________________________________________________________________________________________________________________________________________________________________________________________________________________________________________________________________________________________ | | | | | | | | |  |

| ***What could be disadvantages for you of the Tobacco ROI tool?  I belief that a tool such as the Tobacco ROI tool:*** | Strongly Disagree | | | Strongly  Agree | | | |  |
| --- | --- | --- | --- | --- | --- | --- | --- | --- |
| 1. is too time consuming to use | 1 | 2 | 3 | 4 | 5 | 6 | 7 | ? |
| 1. is not useful for my organisation | 1 | 2 | 3 | 4 | 5 | 6 | 7 | ? |
| 1. requires too much data input | 1 | 2 | 3 | 4 | 5 | 6 | 7 | ? |
| 1. is too complex to work with | 1 | 2 | 3 | 4 | 5 | 6 | 7 | ? |
| 1. is not compatible with the way we work in our organisation | 1 | 2 | 3 | 4 | 5 | 6 | 7 | ? |
| 1. provides unreliable outcomes | 1 | 2 | 3 | 4 | 5 | 6 | 7 | ? |
| 1. does not provide insight on how the results are calculated | 1 | 2 | 3 | 4 | 5 | 6 | 7 | ? |
| 1. results into outcomes that are too broad | 1 | 2 | 3 | 4 | 5 | 6 | 7 | ? |
| 1. is not needed for my daily work | 1 | 2 | 3 | 4 | 5 | 6 | 7 | ? |
| 1. What other disadvantages would a tool such as the Tobacco ROI tool have for you?   ____________________________________________________________________________________________________________________________________________________________________________________________________________________________________________________________________________________________________________________________________________________________________ | | | | | | | |  |

1. Could you please indicate on a scale from 1 to 7 - 1 meaning “strongly disagree” and 7 meaning “strongly agree” to what extent you agree with the following statements? Please circle your answer in the column to the right.

|  | Strongly Disagree | | | Strongly  Agree | | | |  |
| --- | --- | --- | --- | --- | --- | --- | --- | --- |
| 1. My boss will support me in using a tool such as the Tobacco ROI tool in decision making/information gathering | 1 | 2 | 3 | 4 | 5 | 6 | 7 | ? |
| 1. My other colleagues will support me in using a tool such as the Tobacco ROI tool | 1 | 2 | 3 | 4 | 5 | 6 | 7 | ? |
| 1. My organisation will support me in using a tool such as the Tobacco ROI tool in decision making/information gathering | 1 | 2 | 3 | 4 | 5 | 6 | 7 | ? |
| 1. Reimbursement agencies (e.g. health insurances) will support me in using a tool such as the Tobacco ROI tool in decision making/information gathering | 1 | 2 | 3 | 4 | 5 | 6 | 7 | ? |
| 1. My ministry of health will support me in using a tool such as the Tobacco ROI tool in decision making/information gathering | 1 | 2 | 3 | 4 | 5 | 6 | 7 | ? |
| 1. Health professionals (i.e. GPs, medical specialists) will support me in using a tool such as the Tobacco ROI tool in decision making/information gathering | 1 | 2 | 3 | 4 | 5 | 6 | 7 | ? |
| 1. Who else would support you in using the Tobacco ROI tool?   ____________________________________________________________________________________________________________________________________________________________________________________________________________________________________________________________________________________________________________________________________________________________________________ | | | | | | | |  |
| 1. I would encounter resistance using the Tobacco ROI tool | 1 | 2 | 3 | 4 | 5 | 6 | 7 | ? |
| 1. Which organisations or colleagues would not support you to use the Tobacco ROI tool?   ______________________________________________________________________________________________________________________________________________________________________________________ | | | | | | | |  |
|  | | | | | | | |  |
| **How confident are you about using the Tobacco ROI tool?**  **I am confident that:** | Strongly Disagree | | | Strongly  Agree | | | |  |
| 1. I will be able to use the ROI tool | 1 | 2 | 3 | 4 | 5 | 6 | 7 | ? |
| 1. I will be able to use the ROI tool when I need to enter the information on smoking cessation effects of programs myself | 1 | 2 | 3 | 4 | 5 | 6 | 7 | ? |
| 1. I will be able to understand the ROI tool when the data is given in a list of figures | 1 | 2 | 3 | 4 | 5 | 6 | 7 | ? |
| 1. I will be able to use the ROI tool even if my organisation does not support me | 1 | 2 | 3 | 4 | 5 | 6 | 7 | ? |
| 1. I will be able to use the ROI tool without help from others | 1 | 2 | 3 | 4 | 5 | 6 | 7 | ? |
| 1. Persons such as myself in my organisation will be able to use the ROI tool | 1 | 2 | 3 | 4 | 5 | 6 | 7 | ? |
| 1. I will be able to interpret the output of the ROI tool | 1 | 2 | 3 | 4 | 5 | 6 | 7 | ? |
| 1. I will be able to use the tool with limited knowledge about health economics | 1 | 2 | 3 | 4 | 5 | 6 | 7 | ? |
| 1. I will be able to use the ROI tool without technical support | 1 | 2 | 3 | 4 | 5 | 6 | 7 | ? |
| 1. I will be able to use the ROI tool without a manual | 1 | 2 | 3 | 4 | 5 | 6 | 7 | ? |
| 1. I will be able to use the ROI tool without a website that explains how the ROI tool works | 1 | 2 | 3 | 4 | 5 | 6 | 7 | ? |
| 1. What other difficulties would using a tool such as the Tobacco ROI tool have for you?   ____________________________________________________________________________________________________________________________________________________________________________________________________________________________________________________________________________________________________________________________________________________________________________ | | | | | | | |  |

1. Could you please indicate on a scale from 1 to 7 - 1 meaning “strongly disagree” and 7 meaning “strongly agree” to what extent you agree with the following statements? Please circle your answer in the column to the right.

|  | Strongly Disagree | | | Strongly  Agree | | | |  |
| --- | --- | --- | --- | --- | --- | --- | --- | --- |
| 1. I have the intention to use a tool such as the Tobacco ROI tool | 1 | 2 | 3 | 4 | 5 | 6 | 7 | ? |
| 1. I have the intention to use a tool such as the Tobacco ROI tool within the next month | 1 | 2 | 3 | 4 | 5 | 6 | 7 | ? |
| 1. I have the intention to use a tool such as the Tobacco ROI tool within the next 6 months | 1 | 2 | 3 | 4 | 5 | 6 | 7 | ? |
| 1. I have the intention to use a tool such as the Tobacco ROI tool within the next year | 1 | 2 | 3 | 4 | 5 | 6 | 7 | ? |
| 1. I would like to have more information about the Tobacco ROI tool | 1 | 2 | 3 | 4 | 5 | 6 | 7 | ? |

**Part D. Availability of tobacco control measures & smoking cessation interventions**

1. Please read this table! Would you tell me whether these tobacco control measures and smoking cessation interventions are available in ………? Could you also please indicate on a scale from 1 to 3 - 1 meaning “not important” and 3 meaning “important” - to what extent you think the following interventions are considered important in addressing smoking behaviour?

Note that the table proceeds on the next page.

| **Category** | **Name of intervention** | **Availability** | | | **Importance** | | | |
| --- | --- | --- | --- | --- | --- | --- | --- | --- |
| Pharmacological | Nicotine replacement therapy | Yes | No | ? | 1 | 2 | 3 | ? |
|  | Bupropion | Yes | No | ? | 1 | 2 | 3 | ? |
|  | Varinicline | Yes | No | ? | 1 | 2 | 3 | ? |
| Behavioural | Brief advice on smoking cessation given during one GP consultation | Yes | No | ? | 1 | 2 | 3 | ? |
|  | Advice on smoking cessation given according to the 5-step protocol (minimal intervention) | Yes | No | ? | 1 | 2 | 3 | ? |
|  | Community pharmacy-based services | Yes | No | ? | 1 | 2 | 3 | ? |
|  | Computer tailored programs | Yes | No | ? | 1 | 2 | 3 | ? |
|  | Internet based interventions | Yes | No | ? | 1 | 2 | 3 | ? |
|  | Group counselling by specially trained professionals | Yes | No | ? | 1 | 2 | 3 | ? |
|  | Individual counselling by specially trained professionals | Yes | No | ? | 1 | 2 | 3 | ? |
|  | Telephone counselling | Yes | No | ? | 1 | 2 | 3 | ? |
|  | Self-help manuals | Yes | No | ? | 1 | 2 | 3 | ? |
|  | Mobile phone-based interventions | Yes | No | ? | 1 | 2 | 3 | ? |
|  | Stage-based interventions | Yes | No | ? | 1 | 2 | 3 | ? |
| Combined | Individual counselling by specially trained professionals with medication (e.g. NRT or bupropion) | Yes | No | ? | 1 | 2 | 3 | ? |
|  | Group counselling by specially trained professionals with medication (e.g. NRT or bupropion) | Yes | No | ? | 1 | 2 | 3 | ? |
|  | Brief advice by GP and medication | Yes | No | ? | 1 | 2 | 3 | ? |
| Nonconventional | Acupuncture | Yes | No | ? | 1 | 2 | 3 | ? |
|  | Aromatherapy | Yes | No | ? | 1 | 2 | 3 | ? |
|  | Herbs | Yes | No | ? | 1 | 2 | 3 | ? |
|  | Smokeless tobacco | Yes | No | ? | 1 | 2 | 3 | ? |
|  | Hypnosis-based interventions | Yes | No | ? | 1 | 2 | 3 | ? |
|  | Magnetic resonance therapy | Yes | No | ? | 1 | 2 | 3 | ? |
|  | Homeopathy | Yes | No | ? | 1 | 2 | 3 | ? |
| Preventative | Advertising restrictions/bans | Yes | No | ? | 1 | 2 | 3 | ? |
|  | Product labelling and information/ Health warnings on tobacco products | Yes | No | ? | 1 | 2 | 3 | ? |
|  | Restrictions on sales to minors | Yes | No | ? | 1 | 2 | 3 | ? |
|  | Restrictions on smoking in workplaces and public places | Yes | No | ? | 1 | 2 | 3 | ? |
|  | Mass media campaigns | Yes | No | ? | 1 | 2 | 3 | ? |
|  | Tax increase | Yes | No | ? | 1 | 2 | 3 | ? |

1. What would you like to get out of being involved in this research project for you and your organisation?

_________________________________________________________________________________________________________________________________________________________________________________________________________________________________________________________________________________

1. The current version of the ROI model includes the following smoking-related diseases: lung cancer, coronary heart disease, chronic obstructive pulmonary disease, myocardial infarction, and stroke. Are there any other smoking related diseases that you would include in the ROI tool?

_________________________________________________________________________________________________________________________________________________________________________________________________________________________________________________________________________________

1. Comments about the interview:

____________________________________________________________________________________________________________________________________________________________________________________________________________________________________________________________________________________________________________________________________________________________________________

1. See definition of stakeholder in section 2. [↑](#footnote-ref-1)
