# Supplementary material for: Understanding perceived availability and importance of tobacco control interventions to inform European adoption of a UK economic model: a cross-sectional study
Source: BMC Health Serv Res. 2018 Feb 14;18:115. doi: 10.1186/s12913-018-2923-2 (PMC5813331; doi:10.1186/s12913-018-2923-2)
Supplement: Supplementary file 2 — Table S1. Distribution of stakeholders in each country. Table S2. Perceived availability and importance of tobacco-control interventions across countries. (DOC 278 kb) [file 12913_2018_2923_MOESM2_ESM.doc]

**Additional file 2**

**Table S1 Distribution of stakeholders in each country**

|  | **Decision makers** | **Purchasers of services/pharmaceutical products** | **Providers of services** | **Evidence generators** | **Health-promotion advocates** |
| --- | --- | --- | --- | --- | --- |
| **Germany** | 3 (17.7%)^a^ | 0 (0%) | 5 (29.4%) | 3 (17.7%) | 6 (35.3%) |
| **Hungary** | 3 (18.8%) | 2 (12.5%) | 3 (18.8%) | 6 (37.5%) | 2 (12.5%) |
| **The Netherlands** | 8 (28.6%) | 2 (7.1%) | 4 (14.3%) | 11 (39.3%) | 3 (10.7%) |
| **Spain** | 6 (33.3%) | 1 (5.6%) | 5 (27.8%) | 4 (22.2%) | 2 (11.1%) |
| **The United Kingdom** | 9 (64.3%) | 2 (14.3%) | 1 (7.1%) | 1 (7.1%) | 1 (7.1%) |

^a^ Number (Row percentage)

**Table S2** Perceived availability and importance of tobacco-control interventions across countries

|  | Germany  (N = 17) | | Hungary  (N = 16) | | The Netherlands  (N = 28) | | Spain  (N = 18) | | UK  (N = 14) | | Total  (N = 93) | |
| --- | --- | --- | --- | --- | --- | --- | --- | --- | --- | --- | --- | --- |
|  | Availability | Importance | Availability | Importance | Availability | Importance | Availability | Importance | Availability | Importance | Availability | Importance |
| **Pharmacological** | | | | | | | | | | | |  |
| **Nicotine replacement therapy** | | | | | | | | | | | |  |
|  | **% A 100** | % I 58.8 | **% A 100** | % I 87.5 | **% A 96.4** | % I 75.0 | **% A 88.9** | % I 72.2 | **% A 100** | % I 92.9 | **% A 96.8** | % I 76.3 |
|  | **% N 0** | % S 29.4 | **% N 0** | % S 6.3 | **% N 0** | % S 21.4 | **% N 11.1** | % S 16.7 | **% N 0** | % S 7.1 | **% N 2.2** | % S 17.2 |
|  | **% DK 0** | % NI 11.8 | **% DK 0** | % NI 0 | **% DK 3.6** | % NI 0 | **% DK 0** | % NI 11.1 | **% DK 0** | % NI 0 | **% DK 1.1** | % NI 4.3 |
|  |  | % DK 0 |  | % DK 6.3 |  | % DK 3.6 |  | % DK 0 |  | % DK 0 |  | % DK 2.2 |
| **Bupropion** | | | | | | | | | | | |  |
|  | **% A 58.8** | % I 5.9 | **% A 31.3** | % I 43.8 | **% A 64.3** | % I 35.7 | **% A 83.3** | % I 38.9 | **% A 92.9** | % I 71.4 | **% A 65.6** | % I 37.6 |
|  | **% N 0** | % S 29.4 | **% N 18.8** | % S 6.3 | **% N 0** | % S 21.4 | **% N 11.1** | % S 33.3 | **% N 0** | % S 21.4 | **% N 5.4** | % S 22.6 |
|  | **%DK 41.2** | % NI 17.7 | **% DK 50.0** | % NI 0 | **% DK 35.7** | % NI 7.1 | **% DK 5.6** | % NI 22.2 | **% DK 7.1** | % NI 0 | **% DK 29.0** | % NI 9.7 |
|  |  | % DK 47.1 |  | % DK 50.0 |  | % DK 35.7 |  | % DK 5.6 |  | % DK 7.1 |  | % DK 30.1 |
| **Varenicline** | | | | | | | | | | | |  |
|  | **% A 58.8** | % I 5.9 | **% A 43.8** | % I 43.8 | **% A 57.1** | % I 35.7 | **% A 83.3** | % I 50.0 | **% A 92.9** | % I 100 | **% A 65.6** | % I 44.1 |
|  | **% N 0** | % S 35.3 | **% N 0** | % S 0 | **% N 0** | % S 17.9 | **% N 5.6** | % S 22.2 | **% N 0** | % S 0 | **% N 1.1** | % S 16.1 |
|  | **% DK 41.2** | % NI 11.8 | **% DK 56.3** | % NI 0 | **% DK 42.9** | % NI 3.6 | **% DK 11.1** | % NI 11.1 | **% DK 7.1** | % NI 0 | **% DK 33.3** | % NI 5.4 |
|  |  | % DK 47.1 |  | % DK 56.3 |  | % DK 42.9 |  | % DK 16.7 |  | % DK 0 |  | % DK 34.4 |
| **Behavioral** | | | | | | | | | | | |  |
| **Brief advice on smoking cessation given during one GP consultation** | | | | | | | | | | | |  |
|  | **% A 100** | % I 64.7 | **% A 93.8** | % I 93.8 | **% A 100** | % I 75.0 | **% A 100** | % I 77.8 | **% A 85.7** | % I 92.9 | **% A 96.8** | % I 79.6 |
|  | **% N 0** | % S 11.8 | **% N 6.3** | % S 6.3 | **% N 0** | % S 7.1 | **% N 0** | % S 22.2 | **% N 0** | % S 7.1 | **% N 1.1** | % S 10.8 |
|  | **% DK 0** | % NI 23.5 | **% DK 0** | % NI 0 | **% DK 0** | % NI 17.9 | **% DK 0** | % NI 0 | **% DK 14.3** | % NI 0 | **% DK 2.2** | % NI 9.7 |
|  |  | % DK 0 |  | % DK 0 |  | % DK 0 |  | % DK 0 |  | % DK 0 |  | % DK 0 |
| **Advice on smoking cessation given according to the 5-step protocol (minimal intervention)** | | | | | | | | | | | |  |
|  | **% A 82.4** | % I 58.8 | **% A 87.5** | % I 87.5 | **% A 85.7** | % I 71.4 | **% A 72.2** | % I 66.7 | **% A 64.3** | % I 71.4 | **% A 79.6** | % I 71.0 |
|  | **% N 5.9** | % S 23.5 | **% N 0** | % S 0 | **% N 0** | % S 14.3 | **% N 16.7** | % S 16.7 | **% N 0** | % S 7.1 | **% N 4.3** | % S 12.9 |
|  | **% DK 11.8** | % NI 11.8 | **% DK 12.5** | % NI 0 | **% DK 14.3** | % NI 0 | **% DK 11.1** | % NI 5.6 | **% DK 35.7** | % NI 7.1 | **% DK 16.1** | % NI 4.3 |
|  |  | % DK 5.9 |  | % DK 12.5 |  | % DK 14.3 |  | % DK 11.1 |  | % DK 14.3 |  | % DK 11.8 |
| **Community pharmacy-based services** | | | | | | | | | | | |  |
|  | **% A 76.5** | % I 29.4 | **% A 75.0** | % I 62.5 | **% A 53.6** | % I 10.7 | **% A 50.0** | % I 33.3 | **% A 92.9** | % I 85.7 | **% A 66.7** | % I 38.7 |
|  | **% N 5.9** | % S 23.5 | **% N 18.8** | % S 18.8 | **% N 14.3** | % S 28.6 | **% N 38.9** | % S 33.3 | **% N 0** | % S 14.3 | **% N 16.1** | % S 24.7 |
|  | **% DK 17.7** | % NI 35.3 | **% DK 6.3** | % NI 6.3 | **% DK 32.1** | % NI 32.1 | **% DK 11.1** | % NI 16.7 | **% DK 7.1** | % NI 0 | **% DK 17.2** | % NI 20.4 |
|  |  | % DK 11.8 |  | % DK 12.5 |  | % DK 28.6 |  | % DK 16.7 |  | % DK 0 |  | % DK 16.1 |
| **Computer tailored programs** | | | | | | | | | | | |  |
|  | **% A 88.2** | % I 17.7 | **% A 43.8** | % I 37.5 | **% A 85.7** | % I 32.1 | **% A 55.6** | % I 22.2 | **% A 42.9** | % I 42.9 | **% A 66.7** | % I 30.1 |
|  | **% N 5.9** | % S 64.7 | **% N 12.5** | % S 12.5 | **% N 0** | % S 42.9 | **% N 33.3** | % S 27.8 | **% N 21.4** | % S 28.6 | **% N 12.9** | % S 36.6 |
|  | **% DK 5.9** | % NI 11.8 | **% DK 43.8** | % NI 6.3 | **% DK 14.3** | % NI 17.9 | **% DK 11.1** | % NI 22.2 | **% DK 35.7** | % NI 7.1 | **% DK 20.4** | % NI 14.0 |
|  |  | % DK 5.9 |  | % DK 43.8 |  | % DK 7.1 |  | % DK 27.8 |  | % DK 21.4 |  | % DK 19.4 |
| **Internet based interventions** | | | | | | | | | | | |  |
|  | **% A 88.2** | % I 17.7 | **% A 75.0** | % I 50.0 | **% A 92.9** | % I 53.6 | **% A 38.9** | % I 16.7 | **% A 64.3** | % I 50.0 | **% A 74.2** | % I 38.7 |
|  | **% N 0** | % S 64.7 | **% N 6.3** | % S 18.8 | **% N 0** | % S 21.4 | **% N 44.4** | % S 33.3 | **% N 14.3** | % S 28.6 | **% N 11.8** | % S 32.3 |
|  | **% DK 11.8** | % NI 11.8 | **% DK 18.8** | % NI 12.5 | **% DK 7.1** | % NI 14.3 | **% DK 16.7** | % NI 16.7 | **% DK 21.4** | % NI 0 | **% DK 14.0** | % NI 11.8 |
|  |  | % DK 5.9 |  | % DK 18.8 |  | % DK 10.7 |  | % DK 33.3 |  | % DK 21.4 |  | % DK 17.2 |
| **Group counselling by specially trained professionals** | | | | | | | | | | | |  |
|  | **% A 100** | % I 88.2 | **% A 87.5** | % I 62.5 | **% A 100** | % I 78.6 | **% A 72.2** | % I 72.2 | **% A 100** | % I 100 | **% A 92.5** | % I 79.6 |
|  | **% N 0** | % S 11.8 | **% N 0** | % S 18.8 | **% N 0** | % S 21.4 | **% N 27.8** | % S 22.2 | **% N 0** | % S 0 | **% N 5.4** | % S 16.1 |
|  | **% DK 0** | % NI 0 | **% DK 12.5** | % NI 12.5 | **% DK 0** | % NI 0 | **% DK 0** | % NI 5.6 | **% DK 0** | % NI 0 | **% DK 2.2** | % NI 3.2 |
|  |  | % DK 0 |  | % DK 6.3 |  | % DK 0 |  | % DK 0 |  | % DK 0 |  | % DK 1.1 |
| **Individual counselling by specially trained professionals** | | | | | | | | | | | |  |
|  | **% A 100** | % I 82.4 | **% A 87.5** | % I 93.8 | **% A 92.9** | % I 75.0 | **% A 72.2** | % I 61.1 | **% A 100** | % I 85.7 | **% A 90.3** | % I 78.5 |
|  | **% N 0** | % S 11.8 | **% N 0** | % S 0 | **% N 0** | % S 21.4 | **% N 27.8** | % S 16.7 | **% N 0** | % S 14.3 | **% N 5.4** | % S 14.0 |
|  | **% DK 0** | % NI 5.9 | **% DK 12.5** | % NI 0 | **% DK 7.1** | % NI 0 | **% DK 0** | % NI 11.1 | **% DK 0** | % NI 0 | **% DK 4.3** | % NI 3.2 |
|  |  | % DK 0 |  | % DK 6.3 |  | % DK 3.6 |  | % DK 11.1 |  | % DK 0 |  | % DK 4.3 |
| **Telephone counselling** | | | | | | | | | | | |  |
|  | **% A 100** | % I 29.4 | **% A 68.8** | % I 68.8 | **% A 92.9** | % I 35.7 | **% A 55.6** | % I 27.8 | **% A 85.7** | % I 64.3 | **% A 81.7** | % I 43.0 |
|  | **% N 0** | % S 52.9 | **% N 0** | % S 18.8 | **% N 0** | % S 53.6 | **% N 38.9** | % S 27.8 | **% N 0** | % S 28.6 | **% N 7.5** | % S 38.7 |
|  | **% DK 0** | % NI 17.7 | **% DK 31.3** | % NI 0 | **% DK 7.1** | % NI 3.6 | **% DK 5.6** | % NI 27.8 | **% DK 14.3** | % NI 0 | **% DK 10.8** | % NI 9.7 |
|  |  | % DK 0 |  | % DK 12.5 |  | % DK 7.1 |  | % DK 16.7 |  | % DK 7.1 |  | % DK 8.6 |
| **Self-help manuals** | | | | | | | | | | | |  |
|  | **% A 100** | % I 29.4 | **% A 93.8** | % I 50.0 | **% A 92.9** | % I 21.4 | **% A 88.9** | % I 38.9 | **% A 71.4** | % I 35.7 | **%A 90.3** | % I 33.3 |
|  | **% N 0** | % S 29.4 | **% N 0** | % S 25.0 | **% N 3.6** | % S 46.4 | **% N 11.1** | % S 38.9 | **% N 7.1** | % S 42.9 | **% N 4.3** | % S 37.6 |
|  | **% DK 0** | % NI 41.2 | **% DK 6.3** | % NI 18.8 | **% DK 3.6** | % NI 32.1 | **% DK 0** | % NI 16.7 | **% DK 21.4** | % NI 14.3 | **% DK 5.4** | % NI 25.8 |
|  |  | % DK 0 |  | % DK 6.3 |  | % DK 0 |  | % DK 5.6 |  | % DK 7.1 |  | % DK 3.2 |
| **Mobile phone-based interventions** | | | | | | | | | | | |  |
|  | **% A 100** | % I 11.8 | **% A 43.8** | % I 56.3 | **% A 78.6** | % I 25.0 | **% A 22.2** | % I 5.6 | **% A 64.3** | % I 35.7 | **% A 63.4** | % I 25.8 |
|  | **% N 0** | % S 64.7 | **% N 12.5** | % S 18.8 | **% N 3.6** | % S 42.9 | **% N 66.7** | % S 33.3 | **% N 14.3** | % S 50.0 | **% N 18.3** | % S 41.9 |
|  | **% DK 0** | % NI 23.5 | **% DK 43.8** | % NI 12.5 | **% DK 17.9** | % NI 14.3 | **% DK 11.1** | % NI 16.7 | **% DK 21.4** | % NI 0 | **% DK 18.3** | % NI 14.0 |
|  |  | % DK 0 |  | % DK 12.5 |  | % DK 17.9 |  | % DK 44.4 |  | % DK 14.3 |  | % DK 18.3 |
| **Stage-based interventions** | | | | | | | | | | | |  |
|  | **% A 88.2** | % I 35.3 | **% A 43.8** | % I 68.8 | **% A 64.3** | % I 21.4 | **% A 66.7** | % I 55.6 | **% A 57.1** | % I 42.9 | **% A 64.5** | % I 41.9 |
|  | **% N 0** | % S 29.4 | **% N 6.3** | % S 0 | **% N 3.6** | % S 32.1 | **% N 11.1** | % S 16.7 | **% N 14.3** | % S 14.3 | **% N 6.5** | % S 20.4 |
|  | **% DK 11.8** | % NI 17.7 | **% DK 50.0** | % NI 18.8 | **% DK 32.1** | % NI 7.1 | **% DK 22.2** | % NI 11.1 | **% DK 28.6** | % NI 14.3 | **% DK 29.0** | % NI 12.9 |
|  |  | % DK 17.7 |  | % DK 12.5 |  | % DK 39.3 |  | % DK 16.7 |  | % DK 28.6 |  | % DK 24.7 |
| **Combined** | | | | | | | | | | | |  |
| **Individual counselling by specially trained professionals with medication (e.g. NRT or bupropion)** | | | | | | | | | | | |  |
|  | **% A 100** | % I 88.2 | **% A 100** | % I 100 | **% A 89.3** | % I 75.0 | **% A 88.9** | % I 77.8 | **% A 100** | % I 100 | **% A 94.6** | % I 86.0 |
|  | **% N 0** | % S 5.9 | **% N 0** | % S 0 | **% N 0** | % S 10.7 | **% N 11.1** | % S 16.7 | **% N 0** | % S 0 | **% N 2.2** | % S 7.5 |
|  | **% DK 0** | % NI 5.9 | **% DK 0** | % NI 0 | **% DK 10.7** | % NI 3.6 | **% DK 0** | % NI 5.6 | **% DK 0** | % NI 0 | **% DK 3.2** | % NI 3.2 |
|  |  | % DK 0 |  | % DK 0 |  | % DK 10.7 |  | % DK 0 |  | % DK 0 |  | % DK 3.2 |
| **Group counselling by specially trained professionals with medication (e.g. NRT or bupropion)** | | | | | | | | | | | |  |
|  | **% A 94.1** | % I 70.6 | **% A 87.5** | % I 75.0 | **% A 92.9** | % I 78.6 | **% A 72.2** | % I 77.8 | **% A 100** | % I 100 | **% A 89.3** | % I 79.6 |
|  | **% N 5.9** | % S 17.7 | **% N 0** | % S 18.8 | **% N 0** | % S 10.7 | **% N 27.8** | % S 22.2 | **% N 0** | % S 0 | **% N 6.5** | % S 14.0 |
|  | **% DK 0** | % NI 5.9 | **% DK 12.5** | % NI 0 | **% DK 7.1** | % NI 3.6 | **% DK 0** | % NI 0 | **% DK 0** | % NI 0 | **% DK 4.3** | % NI 2.2 |
|  |  | % DK 5.9 |  | % DK 6.3 |  | % DK 7.1 |  | % DK 0 |  | % DK 0 |  | % DK 4.3 |
| **Brief advice by GP and medication** | | | | | | | | | | | |  |
|  | **% A 100** | % I 29.4 | **% A 81.3** | % I 68.8 | **% A 85.7** | % I 53.6 | **% A 83.3** | % I 55.6 | **% A 92.9** | % I 71.4 | **% A 88.2** | % I 54.8 |
|  | **% N 0** | % S 52.9 | **% N 12.5** | % S 25.0 | **% N 7.1** | % S 25.0 | **% N 16.7** | % S 27.8 | **% N 0** | % S 28.6 | **% N 7.5** | % S 31.2 |
|  | **% DK 0** | % NI 17.7 | **% DK 6.3** | % NI 6.3 | **% DK 7.1** | % NI 17.9 | **% DK 0** | % NI 11.1 | **% DK 7.1** | % NI 0 | **% DK 4.3** | % NI 11.8 |
|  |  | % DK 0 |  | % DK 0 |  | % DK 3.6 |  | % DK 5.6 |  | % DK 0 |  | % DK 2.2 |
| **Non-conventional** | | | | | | | | | | | |  |
| **Acupuncture** | | | | | | | | | | | |  |
|  | **% A 100** | % I 11.8 | **% A 100** | % I 18.8 | **% A 96.4** | % I 0 | **% A 22.2** | % I 0 | **% A 42.9** | % I 7.1 | **% A 75.3** | % I 6.5 |
|  | **% N 0** | % S 41.2 | **% N 0** | % S 25.0 | **% N 0** | % S 10.7 | **% N 61.1** | % S 16.7 | **% N 50.0** | % S 28.6 | **% N 19.4** | % S 22.6 |
|  | **% DK 0** | % NI 47.1 | **% DK 0** | % NI 37.5 | **% DK 3.6** | % NI 82.1 | **% DK 16.7** | % NI 50.0 | **% DK 7.1** | % NI 57.1 | **% DK 5.4** | % NI 58.1 |
|  |  | % DK 0 |  | % DK 18.8 |  | % DK 7.1 |  | % DK 33.3 |  | % DK 7.1 |  | % DK 12.9 |
| **Aromatherapy** | | | | | | | | | | | |  |
|  | **% A 82.4** | % I 0 | **% A 75.0** | % I 6.3 | **% A 64.3** | % I 0 | **% A 5.6** | % I 0 | **% A 21.4** | % I 0 | **% A 51.6** | % I 1.1 |
|  | **% N 5.9** | % S 11.8 | **% N 0** | % S 18.8 | **% N 3.6** | % S 0 | **% N 66.7** | % S 16.7 | **% N 50.0** | % S 7.1 | **% N 22.6** | % S 9.7 |
|  | **% DK 11.8** | % NI 76.5 | **% DK 25.0** | % NI 50.0 | **% DK 32.1** | % NI 78.6 | **% DK 27.8** | % NI 44.4 | **% DK 28.6** | % NI 78.6 | **% DK 25.8** | % NI 66.7 |
|  |  | % DK 11.8 |  | % DK 25.0 |  | % DK 21.4 |  | % DK 38.9 |  | % DK 14.3 |  | % DK 22.6 |
| **Herbs** | | | | | | | | | | | |  |
|  | **% A 82.4** | % I 0 | **% A 93.8** | % I 25.0 | **% A 71.4** | % I 0 | **% A 5.6** | % I 0 | **% A 28.6** | % I 7.1 | **% A 58.1** | % I 5.4 |
|  | **% N 0** | % S 11.8 | **% N 0** | % S 12.5 | **% N 3.6** | % S 0 | **% N 66.7** | % S 11.1 | **% N 57.1** | % S 0 | **% N 22.6** | % S 6.5 |
|  | **% DK 17.7** | % NI 76.5 | **% DK 6.3** | % NI 50.0 | **% DK 25.0** | % NI 75.0 | **% DK 27.8** | % NI 50.0 | **% DK 14.3** | % NI 85.7 | **% DK 19.4** | % NI 67.7 |
|  |  | % DK 11.8 |  | % DK 12.5 |  | % DK 25.0 |  | % DK 38.9 |  | % DK 7.1 |  | % DK 20.4 |
| **Smokeless tobacco** | | | | | | | | | | | |  |
|  | **% A 88.2** | % I 0 | **% A 87.5** | % I 25.0 | **% A 42.9** | % I 0 | **% A 11.1** | % I 0 | **% A 50.0** | % I 7.1 | **% A 53.8** | % I 5.4 |
|  | **% N 11.8** | % S 23.5 | **% N 0** | % S 31.3 | **% N 10.7** | % S 0 | **% N 72.2** | % S 11.1 | **% N 50.0** | % S 7.1 | **% N 26.9** | % S 12.9 |
|  | **% DK 0** | % NI 64.7 | **% DK 12.5** | % NI 37.5 | **% DK 46.4** | % NI 64.3 | **% DK 16.7** | % NI 55.6 | **% DK 0** | % NI 78.6 | **% DK 19.4** | % NI 60.2 |
|  |  | % DK 11.8 |  | % DK 6.3 |  | % DK 35.7 |  | % DK 33.3 |  | % DK 7.1 |  | % DK 21.5 |
| **Hypnosis-based interventions** | | | | | | | | | | | |  |
|  | **% A 100** | % I 11.8 | **% A 75.0** | % I 6.3 | **% A 78.6** | % I 0 | **% A 11.1** | % I 0 | **% A 42.9** | % I 7.1 | **% A 63.4** | % I 4.3 |
|  | **% N 0** | % S 58.8 | **% N 6.3** | % S 31.3 | **% N 3.6** | % S 3.6 | **% N 72.2** | % S 16.7 | **% N 50.0** | % S 21.4 | **% N 23.7** | % S 23.7 |
|  | **% DK 0** | % NI 29.4 | **% DK 18.8** | % NI 31.3 | **% DK 17.9** | % NI 75.0 | **% DK 16.7** | % NI 50.0 | **% DK 7.1** | % NI 64.3 | **% DK 12.9** | % NI 52.7 |
|  |  | % DK 0 |  | % DK 31.3 |  | % DK 21.4 |  | % DK 33.3 |  | % DK 7.1 |  | % DK 19.4 |
| **Magnetic resonance therapy** | | | | | | | | | | | |  |
|  | **% A 82.4** | % I 0 | **% A 87.5** | % I 6.3 | **% A 64.3** | % I 0 | **% A 11.1** | % I 0 | **% A 21.4** | % I 0 | **% A 54.8** | % I 1.1 |
|  | **% N 5.9** | % S 17.7 | **% N 0** | % S 12.5 | **% N 0** | % S 0 | **% N 61.1** | % S 5.6 | **% N 50.0** | % S 0 | **% N 20.4** | % S 6.5 |
|  | **% DK 11.8** | % NI 70.6 | **% DK 12.5** | % NI 62.5 | **% DK 35.7** | % NI 67.9 | **% DK 27.8** | % NI 50.0 | **% DK 28.6** | % NI 78.6 | **% DK 24.7** | % NI 65.6 |
|  |  | % DK 11.8 |  | % DK 18.8 |  | % DK 32.1 |  | % DK 44.4 |  | % DK 21.4 |  | % DK 26.9 |
| **Homeopathy** | | | | | | | | | | | |  |
|  | **% A 94.1** | % I 0 | **% A 100** | % I 12.5 | **% A 75.0** | % I 0 | **% A 5.6** | % I 0 | **% A 42.9** | % I 7.1 | **% A 64.5** | % I 3.2 |
|  | **% N 5.9** | % S 52.9 | **% N 0** | % S 18.8 | **% N 0** | % S 7.1 | **% N 72.2** | % S 11.1 | **% N 50.0** | % S 0 | **% N 22.6** | % S 17.2 |
|  | **% DK 0** | % NI 41.2 | **% DK 0** | % NI 56.3 | **% DK 25.0** | % NI 78.6 | **% DK 22.2** | % NI 50.0 | **% DK 7.1** | % NI 85.7 | **% DK 12.9** | % NI 63.4 |
|  |  | % DK 5.9 |  | % DK 12.5 |  | % DK 14.3 |  | % DK 38.9 |  | % DK 7.1 |  | % DK 16.1 |
| **Population-level** | | | | | | | | | | | |  |
| **Advertising restrictions/bans** | | | | | | | | | | | |  |
|  | **% A 100** | % I 82.4 | **% A 100** | % I 87.5 | **% A 96.4** | % I 71.4 | **% A 88.9** | % I 94.4 | **% A 92.9** | % I 100 | **% A 95.7** | % I 85.0 |
|  | **% N 0** | % S 11.8 | **% N 0** | % S 12.5 | **% N 0** | % S 17.9 | **% N 11.1** | % S 5.6 | **% N 0** | % S 0 | **% N 2.2** | % S 10.8 |
|  | **% DK 0** | % NI 5.9 | **% DK 0** | % NI 0 | **% DK 3.6** | % NI 7.1 | **% DK 0** | % NI 0 | **% DK 7.1** | % NI 0 | **% DK 2.2** | % NI 3.2 |
|  |  | % DK 0 |  | % DK 0 |  | % DK 3.6 |  | % DK 0 |  | % DK 0 |  | % DK 1.1 |
| **Product labelling and information/ Health warnings on tobacco products** | | | | | | | | | | | |  |
|  | **% A 100** | % I 41.2 | **% A 100** | % I 75.0 | **% A 100** | % I 50.0 | **% A 94.4** | % I 66.7 | **% A 92.9** | % I 85.7 | **% A 97.9** | % I 61.3 |
|  | **% N 0** | % S 29.4 | **% N 0** | % S 6.3 | **% N 0** | % S 21.4 | **% N 5.6** | % S 33.3 | **% N 0** | % S 14.3 | **% N 1.1** | % S 21.5 |
|  | **% DK 0** | % NI 29.4 | **% DK 0** | % NI 12.5 | **% DK 0** | % NI 25.0 | **% DK 0** | % NI 0 | **% DK 7.1** | % NI 0 | **% DK 1.1** | % NI 15.1 |
|  |  | % DK 0 |  | % DK 6.3 |  | % DK 3.6 |  | % DK 0 |  | % DK 0 |  | % DK 2.2 |
| **Restrictions on sales to minors** | | | | | | | | | | | | |
|  | **% A 100** | % I 88.2 | **% A 100** | % I 93.8 | **% A 100** | % I 67.9 | **% A 83.3** | % I 83.3 | **% A 92.9** | % I 92.9 | **% A 95.7** | % I 82.8 |
|  | **% N 0** | % S 5.9 | **% N 0** | % S 0 | **% N 0** | % S 14.3 | **% N 16.7** | % S 16.7 | **% N 0** | % S 7.1 | **% N 3.2** | % S 9.7 |
|  | **% DK 0** | % NI 5.9 | **% DK 0** | % NI 6.3 | **% DK 0** | % NI 10.7 | **% DK 0** | % NI 0 | **% DK 7.1** | % NI 0 | **% DK 1.1** | % NI 5.4 |
|  |  | % DK 0 |  | % DK 0 |  | % DK 7.1 |  | % DK 0 |  | % DK 0 |  | % DK 2.2 |
| **Restrictions on smoking in workplaces and public places** | | | | | | | | | | | |  |
|  | **% A 100** | % I 88.2 | **% A 100** | % I 93.8 | **% A 100** | % I 85.7 | **% A 94.4** | % I 100 | **% A 92.9** | % I 92.9 | **% A 97.9** | % I 91.4 |
|  | **% N 0** | % S 5.9 | **% N 0** | % S 0 | **% N 0** | % S 7.1 | **% N 5.6** | % S 0 | **% N 0** | % S 7.1 | **% N 1.1** | % S 4.3 |
|  | **% DK 0** | % NI 5.9 | **% DK 0** | % NI 6.3 | **% DK 0** | % NI 3.6 | **% DK 0** | % NI 0 | **% DK 7.1** | % NI 0 | **% DK 1.1** | % NI 3.2 |
|  |  | % DK 0 |  | % DK 0 |  | % DK 3.6 |  | % DK 0 |  | % DK 0 |  | % DK 1.1 |
| **Mass media campaigns** | | | | | | | | | | | |  |
|  | **% A 94.1** | % I 52.9 | **% A 81.3** | % I 81.3 | **% A 82.1** | % I 46.4 | **% A 50.0** | % I 44.4 | **% A 85.7** | % I 100 | **% A 78.5** | % I 61.3 |
|  | **% N 5.9** | % S 35.3 | **% N 18.8** | % S 12.5 | **% N 10.7** | % S 25.0 | **% N 50.0** | % S 38.9 | **% N 7.1** | % S 0 | **% N 18.3** | % S 23.7 |
|  | **% DK 0** | % NI 5.9 | **% DK 0** | % NI 6.3 | **% DK 7.1** | % NI 25.0 | **% DK 0** | % NI 5.6 | **% DK 7.1** | % NI 0 | **% DK 3.2** | % NI 10.8 |
|  |  | % DK 5.9 |  | % DK 0 |  | % DK 3.6 |  | % DK 11.1 |  | % DK 0 |  | % DK 4.3 |
| **Tax increase** | | | | | | | | | | | |  |
|  | **% A 100** | % I 82.4 | **% A 93.8** | % I 68.8 | **% A 89.3** | % I 78.6 | **% A 72.2** | % I 83.3 | **% A 92.9** | % I 100 | **% A 89.3** | % I 81.7 |
|  | **% N 0** | % S 11.8 | **% N 0** | % S 18.8 | **% N 3.6** | % S 17.9 | **% N 27.8** | % S 11.1 | **% N 0** | % S 0 | **% N 6.5** | % S 12.9 |
|  | **% DK 0** | % NI 5.9 | **% DK 6.3** | % NI 6.3 | **% DK 7.1** | % NI 0 | **% DK 0** | % NI 5.6 | **% DK 7.1** | % NI 0 | **% DK 4.3** | % NI 3.2 |
|  |  | % DK 0 |  | % DK 6.3 |  | % DK 3.6 |  | % DK 0 |  | % DK 0 |  | % DK 2.2 |
| **Kappa coefficient* (Level of agreement)** | **0.1223**  **(Slight)** | 0.2483  (Fair) | **0.1826**  **(Slight)** | 0.2021  (Fair) | **0.1246**  **(Slight)** | 0.2711  (Fair) | **0.2648**  **(Fair)** | 0.2077  (Fair) | **0.2293**  **(Fair)** | 0.4246  (Moderate) | **0.0980**  **(Slight)** | 0.2386  (Fair) |

*Combined Kappa coefficient was calculated from the total number of raters from the respective settings who rated 30 interventions with three ratings of availability (available, not available, and don’t know) and with four ratings of importance (not important, somewhat important, important, and don’t know). All combined kappa coefficients had the p-values less than 0.001.
